# A Novel Neuraminidase-Dependent Hemagglutinin Cleavage Mechanism Enables the Systemic Spread of an H7N6 Avian Influenza Virus

**DOI:** 10.1128/mBio.02369-19

**Published:** 2019-11-05

**Authors:** Hyeok-il Kwon, Young-Il Kim, Su-Jin Park, Eun-Ha Kim, Semi Kim, Young-Jae Si, Min-Suk Song, Philippe Noriel Q. Pascua, Elena A. Govorkova, Robert G. Webster, Richard J. Webby, Young Ki Choi

**Affiliations:** a College of Medicine and Medical Research Institute, Chungbuk National University, Cheongju, Republic of Korea; b Division of Virology, Department of Infectious Diseases, St. Jude Children’s Research Hospital, Memphis, Tennessee, USA; c Zoonotic Infectious Diseases Research Center, Chungbuk National University, Cheongju, Republic of Korea; the Peter Doherty Institute for Infection and Immunity

**Keywords:** H7, N6 neuraminidase, thrombin-like protease, GRG motif, trypsin-independent growth, influenza, pathogenicity, virulence

## Abstract

In this study, we demonstrate a novel mechanism for hemagglutinin (HA) activation in a naturally occurring H7N6 avian influenza A virus strain, A/mallard duck/Korea/6L/2007 (A/Mdk/6L/07). This novel mechanism allows for systemic infection of chickens, ducks, and mice, and A/Mdk/6L/07 can replicate *in vitro* without exogenous trypsin and exhibits broad tissue tropism in animals despite the presence of a monobasic HA cleavage motif (PEIPKGR/G). The trypsin-independent growth phenotype requires the N6 neuraminidase and the specific recognition of glycine at the P2 position of the HA cleavage motif by a thrombin-like protease. Correspondingly, viral growth is significantly attenuated by the addition of a thrombin-like protease inhibitor (argatroban). These data provide evidence for a previously unrecognized virus replication mechanism and support the hypothesis that thrombin-mediated HA cleavage is an important virulence marker and potential therapeutic target for H7 influenza viruses.

## Abstract (precis)

The identification of virulence markers in influenza viruses underpins risk assessment programs and the development of novel therapeutics. The cleavage of the influenza virus HA is a required step in the viral life cycle, and phenotypic differences in viruses can be caused by changes in this process. Here, we describe a novel mechanism for HA cleavage in an H7N6 influenza virus isolated from a mallard duck. The mechanism requires the N6 protein and full activity of thrombin-like proteases and allows the virus to cause systemic infection in chickens, ducks, and mice. The thrombin-mediated cleavage of HA is thus a novel virulence determinant of avian influenza viruses.

## INTRODUCTION

Avian influenza A viruses cause disease not only in birds, but also in humans and other mammals. The H5 and H7 strains of these viruses occasionally develop high pathogenicity (1–4), causing adverse economic effects in the poultry industry. Although influenza A virus can infect many hosts, wild aquatic birds (including ducks, geese, gulls, and shorebirds) are believed to constitute the principal natural reservoir for most virus subtypes (5, 6).

Influenza A viruses possess 2 major glycoproteins, hemagglutinin (HA) and neuraminidase (NA), which protrude from the viral envelope. To infect host cells, the HA protein mediates binding to the cellular receptor, composed of α2,3- or 2,6-sialylated glycans; exposure to low pH in the endosome then drives the virus to fuse with the cellular membrane following cleavage of the precursor HA molecule (HA_0_) into HA_1_ and HA_2_ subunits by cellular proteases (7, 8). Specific cleavage of the HA_0_ precursor protein is required for viral infectivity and is an important determinant of viral tropism and pathogenicity in the infected host.

Typically, the HA proteins of highly pathogenic avian influenza (HPAI) H5 and H7 viruses contain additional basic amino acids (R_X_R/K_R/G) at the cleavage site that can be cleaved by an intracellular subtilisin-type enzyme. In contrast, most low-pathogenic avian influenza (LPAI) viruses have a single arginine at the cleavage site, and the HA of these viruses can be cleaved by trypsin-like proteases, such as type II transmembrane serine proteases (9). Because expression of the trypsin-like proteases is limited to the respiratory tract in mammalian hosts, the replication of LPAI viruses is confined to this target site. Similarly, the requirement for an exogenous trypsin-like protease to support efficient viral replication in cell culture is determined by the nature of the monobasic cleavage site in the HA protein. However, the subtilisin-like proteases that can cleave the HA in HPAI viruses are ubiquitously expressed in many tissues; consequently, these viruses spread systemically in susceptible hosts, and such systemic spread is considered the major determinant for increased virulence (10, 11).

HPAI viruses can also replicate in cultured cells without the need for exogenous trypsin. Additionally, the 1918 pandemic influenza H1N1 virus, which does not harbor a multibasic HA cleavage site, can replicate to high titers in Madin-Darby canine kidney (MDCK) cells without the addition of trypsin; notably, NA is essential for this trypsin-independent growth (11). Similarly, the NA of a related strain, A/WSN/33 (H1N1), also facilitates efficient viral replication in the absence of trypsin by activating serum plasminogen, expanding viral tropism *in vivo* (11). Recently, Tse et al. demonstrated that the HA of an LPAI H9N2 virus contains an R-S-K-R cleavage site that can be cleaved in cells with high levels of furin expression and that a glycosylation site-eliminating mutation in HA allows it to be universally cleaved in cell culture (12). Other molecular mechanisms for trypsin-independent replication of LPAI viruses beyond NA-dependent plasminogen activation remain unclear.

In this study, we report a novel replication mechanism exhibited by a naturally occurring LPAI virus, A/Mallard duck/Korea/6L/2007 (A/Mdk/6L/07 [H7N6]). This virus was isolated from multiple tissues of a dead mallard duck. Despite the virus having many features in common with HPAI viruses, it has maintained a monobasic HA cleavage site. Using several *in vitro* and *in vivo* assays, we provide evidence for a thrombin- and neuraminidase-dependent HA activation mechanism that underpins many of the unique properties of this virus. Thrombin-mediated HA cleavage resulted in wider dissemination of the virus in infected chickens, ducks, and mice; thus, it is a novel virulence determinant of H7 influenza viruses.

## RESULTS

### Genetic characterization of the A/Mdk/6L/07 (H7N6) virus.

During routine surveillance for avian influenza activity in migratory birds, in December 2007, we isolated A/Mdk/6L/07 from multiple organs of a dead mallard duck, including the trachea, lung, spleen, and brain. Whole-genome sequence analysis revealed that virus isolates from multiple tissues of the bird were homologous and of the H7N6 subtype. Therefore, we designated the lung isolate A/Mdk/6L/07. Despite the presence of virus in multiple organs of the duck, deduced amino acid sequence analysis of the HA cleavage site showed that the virus has characteristics associated with an LPAI virus (PEIPKGR/G) and that additional basic amino acids were not present (Table 1). Similarly, an examination of other well-characterized molecular markers did not explain the disseminated nature of the infection.

**TABLE 1:** Comparison of the molecular determinants of the Mdk/Korea/6L/07 (H7N6) avian influenza virus with those of other H7 viruses

Virus	Subtype	Characteristic by protein^*a*^:													
		HA							NA deletion at residue(s):		NS1		PB2 virulence marker for residue:		Expression of PB1-F2 protein
		Cleavage site at residues 334–341	RBS (highly conserved) residue by no:								5-aa deletion (residues 80–84)	Virulence marker in C terminus			
			88	143	174	186	217	219	294	69–73			627	701	
Mdk/Korea/6L/07	H7N6	PEIPKG-R/G	Y	W	H	Y	Q	G	R	No	No	ESEV	E	D	Yes
Ab/Korea/W44/05	H7N3	PEIPKG-R/G	Y	W	H	Y	Q	G	R	No	No	ESEV	E	D	Yes
Mdk/Korea/GH171/07	H7N7	PEIPKG-R/G	Y	W	H	Y	Q	G	R	No	No	ESEV	E	D	Yes
Dk/Korea/A117/10	H7N6	PEIPRG-R/G	Y	W	H	Y	Q	G	R	No	No	ESEV	E	D	Yes
Tk/Italy/9739/02	H7N3	PEIPKG-R/G	Y	W	H	Y	Q	G	R	No	No	ESEV	E	D	Yes
Qu/Aichi/1/09	H7N6	PEIPKR-R/G	Y	W	H	Y	Q	G	R	No	No	GSEV	E	D	Yes
Mdk/Nl/12/00	H7N3	PEIPKG-R/G	Y	W	H	Y	Q	G	R	No	No	ESEV	E	D	Yes
Hu/Nl/219/03	H7N7	PEIPKRRRR/G	Y	W	H	Y	Q	G	R	No	No	ESKV	K	D	Yes

a aa, amino acid.

Sequence analysis of the wider receptor-binding site (RBS) revealed that A/Mdk/6L/07 possessed the highly conserved residues Tyr88, Trp143, His174, and Tyr186 (equivalent to Tyr98, Trp153, His183, and Tyr195 in H3 numbering, respectively), as seen in recent wild bird H7 viruses. No deletions were observed in the NA stalk region, where mutations occur upon virus adaptation in terrestrial birds. A functional PB1-F2 open reading frame was detected, which is typical of viruses of wild bird origin and affects the host defense mechanism, thereby enhancing pathogenicity *in vivo* (13). Amino acid sequence analysis of the NS1 protein revealed that all isolates had a 5-residue deletion at positions 80 to 84, which has been observed in poultry isolates in Hong Kong as far back as 2001 (14). The NS1 C-terminal PDZ-binding motif had the ESEV sequence, which is typical for avian viruses; these motifs are known to confer a severe disease phenotype in mice (15, 16) (Table 1).

All 8 gene segments of the Mdk/Korea/6L/07 virus were related to those of viruses isolated in Asia (South Korea, Japan, and China), and the nucleotide (nt) similarity among these segments was 98.7 to 99.9% (see Table S1 in the supplemental material). Phylogenetic analysis of the HA gene of the Mdk/Korea/6L/07 virus showed that it belongs to the Eurasian lineage and is part of a cluster containing recent H7 avian influenza viruses isolated in South Korea.

10.1128/mBio.02369-19.7 TABLE S1 Sequence homology of each gene of Mdk/Korea/6L/07 (H7N6) influenza virus with reference viruses available in GenBank. Download Table S1, DOCX file, 0.1 MB. Copyright © 2019 Kwon et al. 2019 Kwon et al. This content is distributed under the terms of the Creative Commons Attribution 4.0 International license.

As sequence analyses did not reveal any clues as to the underlying mechanism behind the disseminated nature of the observed infection, we also investigated receptor-binding preferences by performing a solid-phase direct binding assay, as previously described (10, 17). The 2009 pandemic H1N1 virus (A/California/07/2009 [CA/07 {H1N1}]) was used as a positive control for binding to α2,6-linked sialic acids. Although the CA/07 (H1N1) virus exhibited strong affinity for α2,6-linked sialic acids, A/Mdk/6L/07 mainly bound the α2,3-linked sialic acid (Fig. S1). This result confirmed that the A/Mdk/6L/07 virus preferentially binds to avian influenza virus receptors, as suggested by sequence analysis.

10.1128/mBio.02369-19.1 FIG S1 Virus receptor-binding specificity assays. The binding affinity of the inactivated whole viruses to SA α2,3′-SL-PAA-biotin and SA α2,6′-SLN-PAA-biotin glycans are shown. The results shown are the means ± standard deviation (of three replicates). Download FIG S1, PDF file, 0.2 MB. Copyright © 2019 Kwon et al. 2019 Kwon et al. This content is distributed under the terms of the Creative Commons Attribution 4.0 International license.

### A/Mdk/6L/07 (H7N6) virus shows broad tissue tropism in experimentally infected animals.

To determine whether we could experimentally replicate the systemic nature of A/Mdk/6L/07 infection as seen in the original mallard duck, we infected animals oronasally with 10^6.0^ 50% egg infective dose (EID_50_)/ml of A/Mdk/6L/07. For infection, we used 5-week-old, specific-pathogen-free (SPF) White Leghorn chickens (Gallus gallus domesticus) and ducks (Anas platyrhynchos domesticus) and 5-week-old female BALB/c mice. For comparison, animals of all species listed above were infected with a previously isolated LPAI virus, A/aquatic bird/Korea/W44/2005 (H7N3) (A/Ab/W44/05).

A/Mdk/6L/07 replicated in infected chickens and reached a peak viral titer (measured using tracheal swabs) of 4.2 log_10_ EID_50_/ml at 5 days postinfection (dpi), with virus shedding persisting up to 7 dpi (Table 2). Notably, A/Mdk/6L/07 was recovered from the lungs (4.2 log_10_ EID_50_/g), kidneys (1.2 log_10_ EID_50_/g), and heart (1.2 log_10_ EID_50_/g), indicating an ability to replicate outside the respiratory and gastrointestinal tracts (Table 2). In contrast, A/Ab/W44/05 was only detected in the respiratory and intestinal organs where typical LPAI viruses replicate. Although A/Mdk/6L/07 was isolated from a wild mallard duck, virus titers in experimentally infected in ducks were lower than those in chickens; the peak viral titer in tracheal swabs was 2.5 log_10_ EID_50_/ml. However, as seen in the original mallard duck, the virus exhibited broad tissue tropism, as virus was found in the kidney (1.2 log_10_ EID_50_/g), spleen (1.2 log_10_ EID_50_/g), heart (1.5 log_10_ EID_50_/g), and in the respiratory and intestinal organs of infected ducks. The virus, however, replicated to higher titers in the chicken lung than in the duck lung (Table 2). In contrast, A/Ab/W44/05 was only detected in the duck respiratory tract and cloaca. We evaluated the intravenous pathogenicity index (IVPI) of A/Mdk/6L/07 in chickens and found it to be 0.22. Further, depression with decreased food consumption was observed in A/Mdk/6L/07-infected chickens at early time points (1 to 3 dpi), although no death was observed during the test. Although the virus was isolated from fatal cases in migratory mallard ducks, we only observed mild clinical symptoms for 2 to 3 dpi in the experimental setting with SPF birds, suggesting that the Mdk/Korea/6L/07 strain is an LPAI virus. In mice, the 50% mouse lethal dose of A/Mdk/6L/07 is >8.7 log_10_ EID_50_/ml, and this virus induces only mild pathological changes in the lung (Table S2 and Fig. S6). However, A/Mdk/6L/07 could replicate in mice without preadaptation and was detected in the lung, brain, spleen, and heart of infected mice, with peak titers of 4.2 log_10_ EID_50_/ml in lung tissues, showing that its systemic spread was not limited to avian hosts (Table 2). A/Ab/W44/05 was detected only in the lungs (4.2 log_10_ EID_50_/ml at 3 dpi).

**TABLE 2:** Replication and tissue distribution of Mdk/Korea/6L/07 (H7N6) avian influenza viruses in avian and mammalian species

Virus strain by host	Titer (log_10_ EID_50_/g [±SD]) by^*a*^:									
	Day postinfection				Organ^*b*^					
	1	3	5	7	Lung	Brain	Kidney	Spleen	Heart	Cloaca
Chicken^*c*^										
Mdk/Korea/6L/07 (H7N6)	2.5 (0.3)	3.5 (0.5)	4.2 (0.5)	2.2 (0.3)	4.2 (0.5)	-	1.2 (0.3)	-	1.2 (0.3)	1.5 (0.3)
Ab/Korea/W44/05 (H7N3)	3.2 (0.3)	2.2 (0.3)	1.9 (0.3)	-	3.9 (0.3)	-	-	-	-	1.9 (0.3)
Duck^*c*^										
Mdk/Korea/6L/07 (H7N6)	1.5 (0.5)	1.5 (0.5)	2.5 (0.3)	-	2.5 (0.5)	-	1.2 (0.3)	1.2 (0.3)	1.5 (0.5)	2.2 (0.5)
Ab/Korea/W44/05 (H7N3)	1.2 (0.3)	1.5 (0.5)	2.2 (0.3)	-	2.2 (0.3)	-	-	-	-	1.9 (0.3)
Mouse^*d*^										
Mdk/Korea/6L/07 (H7N6)	4.2 (0.5)	2.5 (0.3)	1.5 (0.5)	-	2.5 (0.5)	1.5 (0.5)	-	1.2 (0.5)	2.5 (0.5)	-
Ab/Korea/W44/05 (H7N3)	3.2 (0.3)	4.2 (0.5)	1.5 (0.5)	-	4.2 (0.3)	-	-	-	-	-

a Dashed lines indicate negative for virus detection.

b Virus titration in tissues was performed at 3 dpi.

c Virus titration was based on oropharyngeal swab samples.

d Virus titration was based on lung tissue samples.

10.1128/mBio.02369-19.6 FIG S6 Comparisons of changes in lung histopathology in mice by hematoxylin & eosin (H&E) staining. Lungs from mice infected with each virus were harvested at 3 or 5 dpi. The samples were fixed in 10% neutral-buffered formalin and embedded in paraffin. Histological assessment was conducted using standard hematoxylin and eosin staining and light microscopy (magnification, ×200). The slides were viewed using an Olympus η 71 (Olympus, Tokyo, Japan) microscope and DP controller software to capture images. (A) WT6L. (B) 6L HA C) G338P. (D) 6L HA G338T. (E) 6L HA G338S. (F) 6L HA G338A. (G) 6L HA G338N. (H) 6L HA G338D. (I) 6L+PR8 HA.NA. (J) PR8. (K) PR8 HA S338G. (L) PR8 HAS338G+N6. Download FIG S6, PDF file, 0.8 MB. Copyright © 2019 Kwon et al. 2019 Kwon et al. This content is distributed under the terms of the Creative Commons Attribution 4.0 International license.

10.1128/mBio.02369-19.8 TABLE S2 Viral replication of HA- and NA-substituted viruses in the genetic backbone of 6L virus. Download Table S2, DOCX file, 0.1 MB. Copyright © 2019 Kwon et al. 2019 Kwon et al. This content is distributed under the terms of the Creative Commons Attribution 4.0 International license.

### Trypsin-independent growth of A/Mdk/6L/07 (H7N6) *in vitro*.

As discussed above, an additional characteristic of HPAI viruses is their ability to replicate in cultured cells without exogenous trypsin. Because we had observed systemic spread in infected animals, we wanted to determine whether A/Mdk/6L/07 replication in MDCK cells requires trypsin. For comparison, A/Ab/W44/05 (a negative control) and A/WSN/33 (a positive control) were included.

**FIG 1:**
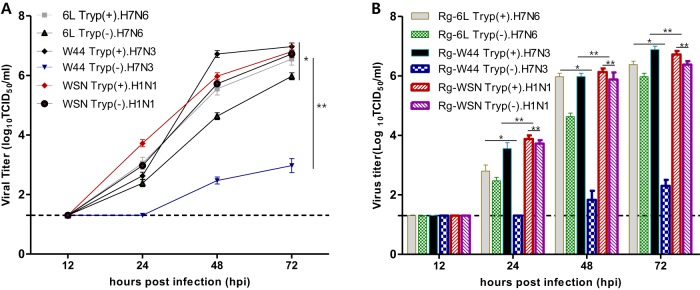
Growth properties of wild-type viruses and viruses generated by reverse genetics in the presence or absence of TPCK-treated trypsin. (A and B) The replication of wild-type viruses in the presence or absence of trypsin (A) and viruses generated by reverse genetics in the presence or absence of trypsin (B) was assessed in MDCK cells at 12, 24, 48, and 72 hours postinfection (hpi). 6L, A/Mdk/6L/07 (H7N6) virus; W44, A/Ab/W44/05 (H7N3); WSN, A/WSN/33 (H1N1); Tryp, TPCK-treated trypsin; +, treated; −, untreated; Rg, reverse genetics. *, *P* < 0.05; **, *P* < 0.01 (Student's *t* test).

A/Mdk/6L/07 was able to replicate to a peak titer of 5.97 log_10_ 50% tissue culture infective dose (TCID_50_)/ml without exogenously added trypsin, although the titers were slightly higher in its presence. In contrast, A/Ab/W44/05 grew poorly (peak titer, 2.97 log_10_ TCID_50_/ml) in the absence of trypsin (Fig. 1). The positive-control virus, A/WSN/33, also replicated in MDCK cells to high titers (6.72 log_10_ TCID_50_/ml) independently of trypsin (Fig. 1).

To prepare to study further the mechanism behind the trypsin-independent growth of A/Mdk/6L/07, we used reverse genetics to generate recombinant versions of A/Mdk/6L/07, A/Ab/W44/05, and A/WSN/33. These recombinant viruses exhibited growth patterns similar to those observed for their respective parental viruses, indicating that wild-type and recombinant viruses shared similar characteristics (Fig. 1B).

### Role of the NA gene of A/Mdk/6L/07 in trypsin-independent viral growth.

**FIG 2:**
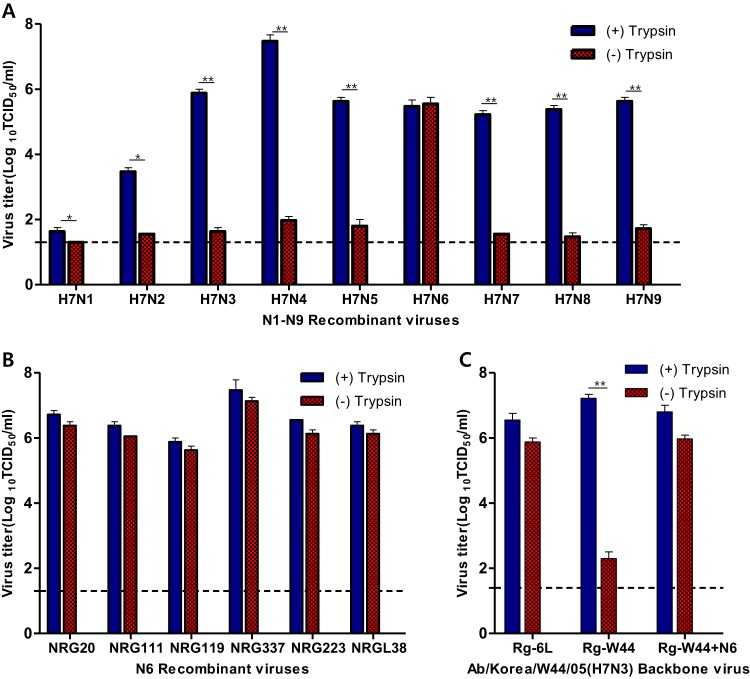
Viral growth properties of recombinant viruses containing different NA genes in the presence or absence of TPCK-treated trypsin *in vitro*. (A) Recombinant viruses were generated with 9 NA genes (N1 through N9) in a backbone of the A/Mdk/6L/07 (H7N6) virus; viral titers were determined in the presence or absence of trypsin. The source of each gene is listed in Table S2. (B) Six H7N6 viruses with N6 genes originating from 6 recently circulating avian influenza strains were generated by using an A/Mdk/6L/07 (H7N6) virus backbone. The growth properties of the viruses were examined in the presence or absence of trypsin. The source of each gene is listed in Table S2. (C) The growth properties of an H7N6 virus in which the N6 gene from the A/Mdk/6L/07 (H7N6) virus was placed in an A/Ab/W44/05 (H7N3) virus backbone were determined in the presence or absence of trypsin. +, treated; −, untreated; Rg, reverse genetics. *, *P* < 0.05; **, *P* < 0.01 (Student's *t* test).

Because the most obvious genetic difference between A/Mdk/6L/07 and A/Ab/W44/05 was the presence of a different NA (N6 and N3, respectively), we hypothesized that N6 was associated with the trypsin-independent growth of A/Mdk/6L/07. To test this hypothesis, recombinant A/Mdk/6L/07-like viruses differing only in their NA genes were generated (Table S3). Of the 9 recombinant viruses (H7N1 through H7N9), only H7N6 replicated to substantial levels in the absence of trypsin (greater than 3.5 log_10_ TCID_50_/ml), indicating that the NA gene (specifically N6) was associated with trypsin-independent viral growth (Fig. 2A).

10.1128/mBio.02369-19.9 TABLE S3 Viral replication of HA- and NA-substituted viruses in the genetic backbone of 6L and PR8 virus. Download Table S3, DOCX file, 0.1 MB. Copyright © 2019 Kwon et al. 2019 Kwon et al. This content is distributed under the terms of the Creative Commons Attribution 4.0 International license.

To determine whether this trait was associated with a specific N6 protein, we generated 6 more A/Mdk/6L/07-like viruses (all H7N6) differing only in the source of their N6 gene (Table S2, various N6 gene panel). All tested viruses reached titers of 5.63 to 7.47 log_10_ TCID_50_/ml with or without exogenous trypsin (Fig. 2B). Transferring the N6 gene from A/Mdk/6L/07 to A/Ab/W44/05 enabled growth in the absence of trypsin (Fig. 2C); A/Ab/W44/05 did not replicate well with its native N3 protein in the absence of trypsin (Fig. 1). Correspondingly, transferring the N3 gene from A/Ab/W44/05 to A/Mdk/6L/07 resulted in the loss of its cytopathic effect 72 h after infection (Fig. S2). These results indicate that N6 plays a critical role in the trypsin-independent viral growth of the tested H7 viruses.

10.1128/mBio.02369-19.2 FIG S2 Cytopathic effect (CPE) of MDCK cells infected with A/Mdk/6L/07 (H7N6) (A) or recombinant H7N3 (B) viruses without trypsin at 24 and 72 hours postinfection. The recombinant H7N3 virus was generated in the backbone of A/Mdk/6L/07, and the N3 gene was isolated from A/Ab/W44/05 (H7N3) virus. Download FIG S2, PDF file, 0.2 MB. Copyright © 2019 Kwon et al. 2019 Kwon et al. This content is distributed under the terms of the Creative Commons Attribution 4.0 International license.

### Genetic analysis of the H7 cleavage site residues suggests the presence of a potentially novel proteolytic motif.

Although we were able to provide evidence that the trypsin-independent growth of A/Mdk/6L/07 is dependent on the N6 gene, the HA protein of this virus, H7, must still be cleaved for efficient growth. Therefore, we explored the genetic characteristics of the cleavage motifs in the HA proteins of H7 viruses by performing single amino acid polymorphism (SAP) analysis of sequences deposited in GenBank and the Influenza Virus Resource (Influenza Virus Resource; Information, Search and Analysis; https://www.ncbi.nlm.nih.gov/genomes/FLU/Database/nph-select.cgi?go=database). SAP analysis revealed that the H7 HA protein contains an invariant arginine at position 339 (H3 numbering), which is the P1 position of the proteolytic cleavage site, and highly conserved proline, lysine, and glycine residues at positions 336, 337, and 338 (the P4, P3, and P2 positions), respectively (Fig. S3A).

10.1128/mBio.02369-19.3 FIG S3 Multiple-sequence alignment of H7 near the proteolytic cleavage site. Molecular analysis and investigation of the protease sensitivity of the HA proteolytic cleavage site and the neighboring regions of the protein were conducted. (A) Amino acid sequence alignment of the HA proteolytic cleavage regions of several H7 and H1 HA proteins. Sequences from the NCBI Influenza Virus Resource (https://www.ncbi.nlm.nih.gov/genomes/FLU/Database/nph-select.cgi?go=database) were aligned by using the Clustal V software, and the aligned HA cleavage site sequences of representative viruses are shown with the corresponding consensus sequence. Dots indicate residues that are identical to consensus residues; the P4-P1′ positions of the cleavage site and the location of the fusion peptide are shown. (B) Variability at the P2 position was associated with the geographic regions of the viruses. (C) Schematic representation of an enzyme-substrate complex with 8 binding sites. Positions Pn to Pm′ in the substrate were determined by counting from the bond between P1 and P1′ (the cleavage site). Download FIG S3, PDF file, 0.1 MB. Copyright © 2019 Kwon et al. 2019 Kwon et al. This content is distributed under the terms of the Creative Commons Attribution 4.0 International license.

The residue at the P2 position correlates with the geographic distribution of each virus (Fig. S3B). Glycine (the dominant residue) was common (39.1%) in Eurasian H7 lineages (found in Italy, Spain, Germany, England, Sweden, the Netherlands, Egypt, South Korea, China, Japan, Hong Kong, Thailand, and other countries), whereas proline (34%), tyrosine (18.9%), and other residues were observed only in American LPAI lineages (the United States, Canada, Chile, Mexico, and New York) or in HPAI H7 isolates (Fig. S3B). We also noted that glycine, arginine, and glycine in positions P2, P1, and P1′, respectively, form an optimal cleavage site for thrombin (Fig. S3C) (18), suggesting that thrombin-like proteases can recognize the HA cleavage motif of H7 viruses, especially those of Eurasian lineages (including A/Mdk/6L/07).

### Effects of HA cleavage motifs on virus replication.

**FIG 3:**
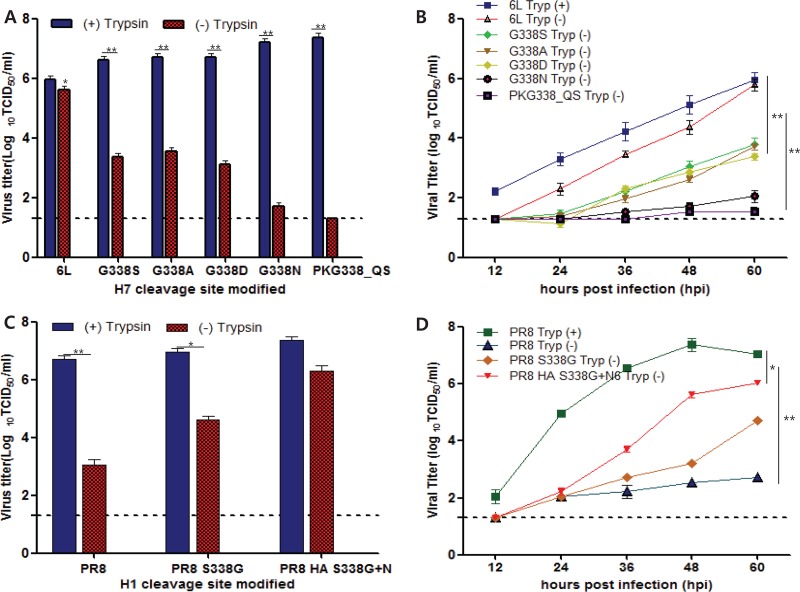
Growth properties and kinetics of viruses modified at the P2 position of the HA cleavage site in the presence or absence of TPCK-treated trypsin. (A) Replication properties of viruses with mutations at the P2 position of A/Mdk/6L/07 (H7N6) HA were observed in the presence or absence of trypsin (*P* < 0.005). 6L, A/Mdk/6L/07 (H7N6) virus. G338S, G338A, G338D, G338N, and PKG338_QS viruses were modified at the indicated positions to the specified amino acids in the genetic backbone of A/Mdk/6L/07 (H7N6); *, *P* < 0.05 (Student's *t* test). Average viral titers are presented above the bar. (B) The growth kinetics of viruses with mutations at the P2 position. G338S, G338A, G338D, G338N, and PKG338_QS viruses were modified at the indicated position to the specified amino acids in the genetic backbone of A/Mdk/6L/07 (H7N6). 6L, A/Mdk/6L/07 (H7N6) virus; Tryp, TPCK-treated trypsin; +, treated; −, untreated. (C and D) Viral titer (C) and replicative kinetics (D) of A/PR/8/34 (H1N1) and of viruses harboring HA and/or NA modifications. PR8 S338G (H1N1) and PR8 S338G+N6 (H1N6) were observed in the absence and presence of trypsin. *, *P* < 0.05; **, *P* < 0.01 (Student's *t* test).

To further investigate the effects of HA cleavage motifs on replication, we modified the native A/Mdk/6L/07 HA cleavage motif (H7_PKGRG_) using PCR-mediated site-directed mutagenesis. We generated recombinant A/Mdk/6L/07 viruses containing serine, alanine, aspartic acid, or asparagine substitutions at the P2 position and a mutant virus (HA_PKG338_QS_) with glutamine and serine substitutions at the P3 and P2 positions, respectively. By doing this, we generated the cleavage motif of the A/PR/8/34 H1N1 virus, which exhibits trypsin-dependent viral growth. Although all of the mutant viruses exhibited similar replication properties in media containing trypsin, a significant reduction in replication was observed in the absence of trypsin (*P* < 0.005; Fig. 3A). The same effect was observed in multicycle replication studies conducted using an MOI of 0.0001 and an incubation time of 60 h (Fig. 3B).

We next replaced the HA cleavage site in A/PR/8/34 with the thrombin motif-containing site from A/Mdk/6L/07 (creating the virus PR8_S338G_). We then made an additional virus by replacing the N1 gene of PR8_S338G_ with the N6 gene (creating the virus PR8_S338G+N6_) (Fig. 3C). The wild-type A/PR/8/34 (H1N1), PR8_S338G_ (H1N1), and PR8_S338G+N6_ (H1N6) viruses replicated efficiently in the presence of trypsin in MDCK cells, indicating that all recombinant PR8 viruses (H1N1 and H1N6) could replicate (Fig. 3C and D). In the absence of trypsin, the PR8 virus replicated only minimally (3.05 log_10_ TCID_50_/ml), PR8_S338G_ replicated to intermediate levels (4.63 log_10_ TCID_50_/ml), and PR8_S338G+N6_ (H1N6) replicated to levels almost comparable to those of the parental virus in the presence of trypsin (7.38 log_10_ TCID_50_/ml). These results demonstrate that the N6 NA and the HA thrombin cleavage sites are necessary and sufficient for trypsin-independent growth in MDCK cells.

To further investigate whether the thrombin cleavage motif was also responsible for systemic infection, as observed in A/Mdk/6L/07-infected mice (Table 2), we infected mice with our panel of recombinant viruses with altered cleavage sites and examined their tissue distribution. As expected, wild-type A/Mdk/6L/07 was recovered from multiple organs, including the brain (1.2 log_10_ EID_50_/0.1 g), spleen (1.2 log_10_ EID_50_/0.1 g), heart (2.2 log_10_ EID_50_/0.1 g), and lungs (3.5 log_10_ EID_50_/0.1 g). None of the recombinant A/Mdk/6L/07 viruses with altered cleavage sites were recovered from any of these organs except the heart; instead, these viruses were primarily detected in the lungs of infected mice (Table 3). In contrast, the PR8_S338G_ and PR8_S338G+N6_ recombinant viruses displayed broad tissue tropism that was expanded (i.e., brain involvement) compared to that of the parental virus. Collectively, these results demonstrate that the HA thrombin cleavage motif operates in concert with N6 to permit expanded tissue tropism in infected mice. The mouse 50% lethal dose (LD_50_) for each wild-type or recombinant virus is reported in Table S2, and the body weight and histopathological results for each virus are shown in Fig. S5 and S6.

**TABLE 3:** Systemic titration of a modified HA cleavage site in Md/Korea/6L/07 (H7N6) in mice

Virus^*b*^	No. of virus detections/total (log_10_ EID_50_/g ± SD) in the^*a*^:						
	Brain	Spleen	Kidney	Intestine	Liver	Heart	Lung
WT6L	3/3 (1.2 ± 0.1)	2/3 (1.2 ± 0.5)	-	-	-	3/3 (2.2 ± 0.1)	3/3 (3.5 ± 0.1)
6L HA G338P	-	-	-	-	-	1/3 (0.4 ± 0.8)	3/3 (4.5 ± 0.1)
6L HA G338T	-	-	-	-	-	-	3/3 (5.5 ± 0.1)
6L HA G338S	-	-	-	-	-	2/3 (1.2 ± 0.7)	3/3 (4.0 ± 0.7)
6L HA G338A	-	-	-	-	-	2/3 (1.2 ± 0.7)	3/3 (3.7 ± 0.1)
6L HA G338N	-	-	-	-	-	-	3/3 (3.4 ± 0.7)
6L HA G338D	-	-	-	-	-	2/3 (1.4 ± 0.7)	3/3 (4.0 ± 0.7)
6L HA PKG338_QS	-	-	-	-	-	1/3 (0.4 ± 0.8)	3/3 (5.2 ± 0.1)
6L+PR8 HA.NA	-	-	-	-	-	-	3/3 (5.2 ± 0.1)
PR8	-	-	-	-	-	2/3 (1.7 ± 0.7)	3/3 (4.0 ± 0.7)
PR8 HA S338G	2/3 (1.2 ± 0.8)	-	-	-	-	3/3 (1.2 ± 0.7)	3/3 (4.7 ± 0.7)
PR8 HA S338G+N6	3/3 (1.2 ± 0.1)	-	-	-	-	2/3 (1.2 ± 0.7)	3/3 (4.4 ± 0.7)

a Dashed lines indicate negative for virus detection.

b Tissue titration was performed at 3 dpi.

10.1128/mBio.02369-19.5 FIG S5 Body weight monitoring following infection of A/Mdk/6L/07 and recombinant viruses in mice. Mice were monitored daily for 14 days following infection with A/Mdk/6L/07 (H7N6) or recombinant viruses, and changes in body weight were noted. Infected mice were euthanized if they lost more than 25% of their initial body weight. Wild-type 6L (WT6L), 6L HA G338P, 6L HA G338T, 6L HA G338S, 6L HA G338A, 6L HA G338N, 6L HA G338D, 6L+PR8 HA.NA, PR8, PR8 HA S338G, and PR8 HAS338G+N6 viruses are shown. Download FIG S5, PDF file, 0.1 MB. Copyright © 2019 Kwon et al. 2019 Kwon et al. This content is distributed under the terms of the Creative Commons Attribution 4.0 International license.

### Impact of the HA subtype on trypsin-independent growth.

Because the cleavage site and NA were important contributors to the trypsin-independent growth of A/Mdk/6L/07, we next wanted to determine the impact of the thrombin cleavage site on the growth of viruses with different HA subtypes. To do so, we created a panel of recombinant A/Mdk/6L/07-based viruses carrying a variety of HA proteins (H1 to H12), each of which was altered to contain the thrombin motif and the N6 NA. We made the same combinations in the A/PR/8/34 virus backbone (Table S4). Of these, the H4, H5, H6, H10, H11, and H12 subtypes were successfully rescued on both backbones and further assayed for growth in MDCK cells with or without exogenous trypsin.

10.1128/mBio.02369-19.10 TABLE S4 Viral titer and MLD_50_ of a modified HA cleavage site in Md/Korea/6L/07 (H7N6). Download Table S4, DOCX file, 0.1 MB. Copyright © 2019 Kwon et al. 2019 Kwon et al. This content is distributed under the terms of the Creative Commons Attribution 4.0 International license.

**FIG 4:**
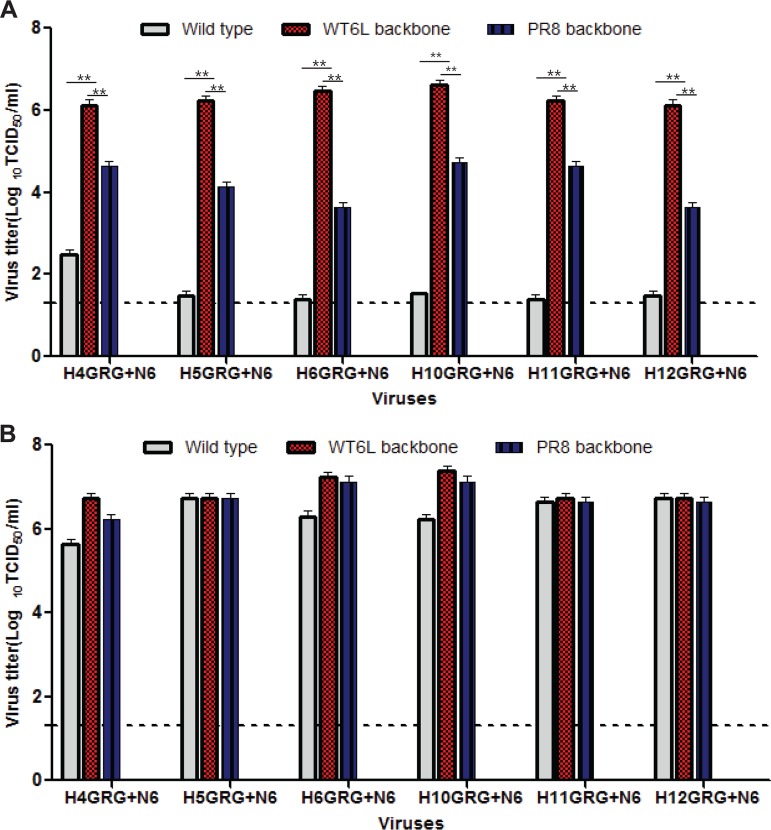
HA_GRG+_N6 from several HA-subtype recombinant viruses contribute to trypsin-independent viral growth in MDCK cells. (A) Wild-type or recombinant viruses that contain a variety of HA_GRG_ and N6 genes were generated by using the A/Mdk/6L/07 (H7N6) or PR8 virus as a backbone. Viral titers were determined in the absence of trypsin. The source of each gene is listed in Table S3. (B) Wild-type or recombinant viruses that contain a variety of HA_GRG_ and N6 genes were generated by using the A/Mdk/6L/07 (H7N6) and PR8 viruses as a backbone. Viral titers were determined in the presence of trypsin. The source of each gene is listed in Table S3. +, treated; −, untreated. *, *P* < 0.05; **, *P* < 0.01 (Student's *t* test).

The 6 A/Mdk/6L/07-based recombinant viruses replicated to substantial levels in the absence of trypsin (greater than 6.13 log_10_ TCID_50_/ml), but their parental viruses exhibited basal levels of replication (lower than 2.47 log_10_ TCID_50_/ml) in the absence of trypsin (Fig. 4 and Table S4). Similarly, all of the A/PR/8/34-based recombinant viruses replicated to titers greater than 3.63 log_10_ TCID_50_/ml in the absence of trypsin, although most of their parental viruses did not reach 2.55 log_10_ TCID_50_/ml in the absence of trypsin (Fig. 4A). As expected, all of the tested viruses exhibited growth patterns that were similar to those of their parental viruses in the presence of trypsin (Fig. 4B).

### Impact of protease inhibitors on virus growth.

Thrombin-like proteases were implicated in the trypsin-independent growth of A/Mdk/6L/07 based solely on the analysis of the HA cleavage site sequence. Therefore, to provide more direct evidence for the contribution of thrombin-like proteases, we assessed the impact of select protease inhibitors on viral growth and HA cleavage patterns. Because aprotinin (a broad-spectrum serine protease inhibitor that inhibits trypsin, plasmin, papain, and cathepsin B but not thrombin) efficiently blocks A/WSN/33 replication in the absence of trypsin (19), we tested whether aprotinin or leupeptin (an inhibitor of trypsin, plasmin, and several other serine proteases) could attenuate the replication of A/Mdk/6L/07 and inhibit the cleavage of HA in the absence of trypsin. We also tested the impact of argatroban (a thrombin-specific inhibitor).

**FIG 5:**
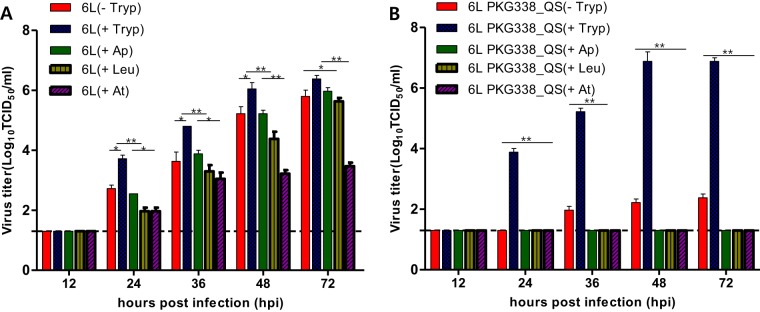
Viral growth kinetics of A/Mdk/6L/07 and recombinant viruses with or without host protease inhibitor and trypsin *in vitro*. The inhibitory effect of protease inhibitors (aprotinin, leupeptin, and argatroban) on the replication of A/Mdk/6L/07 (H7N6) (A) and a modified virus, 6L_PKG338_QS_ (B), containing modified HA cleavage sites in MDCK cells. MDCK cells were infected with A/Mdk/6L/07 (H7N6) and 6L_PKG338_QS_ at an MOI of 0.0001, and samples were collected at 12, 24, 48, and 72 hpi. 6L, A/Mdk/6L/07 (H7N6); 6L PKG338_QS, 6L_PKG338_QS_ virus modified at the indicated position of the specified amino acids by using the A/Mdk/6L/07 (H7N6) virus as a backbone; +, treated; −, untreated. *, *P* < 0.05; **, *P* < 0.01 (Student's *t* test).

The replication of A/Mdk/6L/07 was not attenuated in the presence of aprotinin or leupeptin and exhibited growth kinetics similar to those of A/Mdk/6L/07 in the absence of trypsin (Fig. 5A). Argatroban, the thrombin-specific protease inhibitor, significantly attenuated the growth of A/Mdk/6L/07 (Fig. 5A). In contrast, the HA_PKG338_QS_ recombinant virus grew to levels below the limit of detection when serine protease inhibitors or argatroban were added to the medium, and this virus exhibited normal growth kinetics only in the presence of trypsin (Fig. 5B).

These results demonstrate that the HA cleavage mechanism used by A/Mdk/6L/07 is associated with a thrombin-like protease but differs from that used by the A/WSN/33 virus, the growth of which is affected by broad-spectrum serine protease inhibitors.

### Thrombin activation following A/Mdk/6L/07 infection.

While we were able to show that thrombin-like proteases are important for A/Mdk/6L/07 growth, it was not clear if the virus activated the protein from its inactive precursor form. To assess this, we looked for the activation of thrombin-like proteases during A/Mdk/6L/07 replication in MDCK by Western blotting.

**FIG 6:**
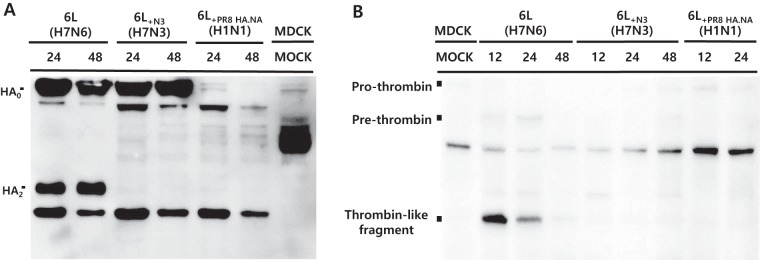
Western blot analysis of HA of A/Mdk/6L/07 and prothrombin cleavage patterns. (A and B) HA of A/Mdk/6L/07 (A) and prothrombin/thrombin (B) cleavage patterns were analyzed with Mdk/Korea/6L/07 (H7N6), recombinant 6L_+N3_ (H7N3), and 6L_+PR8 HA.NA_ (H1N1) virus-infected MDCK cells at 12 to 48 hpi without trypsin conditions. 6L, A/Mdk/6L/07 (H7N6); 6L_+N3_, 6L_+N3_ virus modified at the indicated NA gene by using the A/Ab/W44/05 (H7N3) virus; 6L_+PR8 HA,NA_, 6L_+PR8 HA,NA_ virus modified at the indicated HA and NA genes by using the A/PR/8/34 (H1N1) virus.

Wild-type A/Mdk/6L/07 and recombinant A/Mdk/6L/07 viruses containing the N3 from A/Ab/W44/05 or the HA and NA from A/PR/8/34 were used to infect MDCK cells in the absence of trypsin. Cleavage of A/Mdk/6L/07 HA_0_ into HA_1_ and HA_2_ was observed at 24 and 48 h but was not seen for the recombinant H7N3 and H1N1 viruses (Fig. 6A). Using the same cell lysates, we next sought evidence of cleavage of the prothrombin complex to the active thrombin based on Western blotting with a commercial prothrombin antibody. A 30-kDa band, the expected size of thrombin, was evident in A/Mdk/6L/07-infected cultures but not in cultures of the recombinant viruses (Fig. 6B), suggesting prothrombin activation by the H7N6 virus. To investigate the effect of N6 protein on prothrombin activation, prothrombin protein was incubated with sucrose-purified H7N3 or H7N6 viruses *in vitro* at 37°C for 30 min, and then Western blotting was conducted with prothrombin antibody. Although phosphate-buffered saline (PBS) treatment induced only a basal level of thrombin activation, *in vitro* incubation with H7N6 virus significantly enhanced thrombin activation compared with H7N3 virus (Fig. S4). This result suggests that the N6 protein of H7N6 viruses could directly activate the thrombin by cleavage of the prothrombin during the replication.

10.1128/mBio.02369-19.4 FIG S4 Western blot analysis was conducted to evaluate the activation of prothrombin to pre/thrombin by A/Mdk/6L/07 (H7N6) and the recombinant 6L_+N3_ (H7N3) viruses. 6×-His purified prothrombin (1 μg; Abcam) was incubated with PBS (control), H7N3, or H7N6 viruses *in vitro* for 30 min, and then the cleavage patterns were assessed by Western blotting with His tag monoclonal antibody. PBS, phosphate-buffered saline; H7N3, recombinant 6L_+N3_(H7N3) virus; H7N6, A/Mdk/6L/07 virus. Download FIG S4, PDF file, 0.1 MB. Copyright © 2019 Kwon et al. 2019 Kwon et al. This content is distributed under the terms of the Creative Commons Attribution 4.0 International license.

### Argatroban, a thrombin-specific inhibitor, protects mice from lethal A/Mdk/6L/07 challenge.

**FIG 7:**
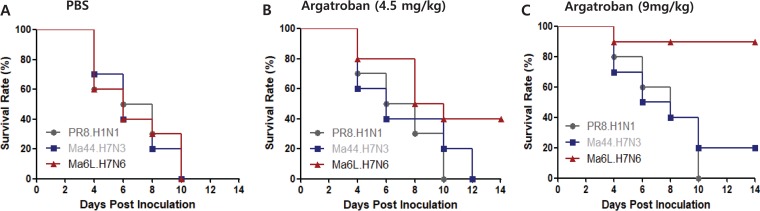
Survival test of A/Mdk/6L/07-inoculated mice treated with host protease inhibitor. The efficacy of argatroban was verified by the survival rate following the lethal influenza virus infection. (A to C) Argatroban at 0 μg (PBS) (A), 67.5 μg (B), and 135 μg (C) in 100 μl was administered intraperitoneally every day for 3 days, followed by intranasal challenge with 5 50% murine lethal doses (MLD_50_s) of PR8.H1N1 (10^3.0^ EID_50_), Ma44.H7N3 (10^4.0^ EID_50_), or Ma6L.H7N6 (10^5.0^ EID_50_) viruses. The survival of mice inoculated with each virus was recorded for 14 dpi.

Because we determined that addition of argatroban was sufficient to block the replication of Mdk/Korea/6L/07 in cultured cells, we next sought to determine its impact *in vivo*. Groups of mice (*n* = 10) were infected with A/PR/8/34 (H1N1), Ab/Korea/Ma44/05 (H7N3) (Ma44), or A/Mdk/6L/07 (H7N6) (6L) virus, and argatroban was administered as indicated (Fig. 7). All PBS control-treated mice died within 10 days, regardless of challenge virus used (Fig. 7A). In the low-dose (4.5 mg/kg) argatroban treatment groups, 6L (H7N6)-infected mice (40%) survived (Fig. 7B). In the high-dose (9 mg/kg) argatroban treatment groups, 9 of 10 6L (H7N6)-infected mice survived with less than 10% of body weight loss. However, no A/PR8/34 (H1N1) virus-infected mice survived, and only 3 of 10 Ma44 (H7N3)-infected mice survived (Fig. 7C).

## DISCUSSION

Here, we report the discovery of a novel replication mechanism used by a naturally occurring LPAI virus, A/Mdk/6L/07, which was isolated from a wild bird. The mechanism relies on a specific NA subtype (N6) and a specific cleavage motif in HA (GRG), and it culminates in altered viral growth *in vitro* and expanded tissue tropism and virulence *in vivo*. Although A/Mdk/6L/07 and A/Ab/W44/05 share a Eurasian H7 HA ancestry (Fig. S3), only A/Mdk/6L/07 replicated in MDCK cells in the absence of trypsin and was recovered from multiple organs of infected birds and mice.

Other than the prototypic HPAI H5 and H7 viruses, only 2 human H1N1 viruses, WSN (H1N1) and the 1918 Spanish influenza virus, have been shown to replicate independently of trypsin and to disseminate to nontarget organs in mice; in the case of the H1N1 viruses, plasminogen was shown to be a critical factor (9, 20). Post et al. reported that certain LPAI viruses, including the H5N2, H7N1, H7N7, and H9N2 subtypes, could systemically infect multiple tissues in infected chickens via an unknown mechanism, whereas the tissue distribution of LPAI viruses in ducks is typically limited to the lungs and to peripheral blood mononuclear cells (21, 22). However, although the molecular properties of A/Mdk/6L/07 were characteristic of low-pathogenicity viruses, the virus was originally isolated from several organs (trachea, lung, spleen, and brain) of a dead mallard duck.

Infection studies in domestic ducks and chickens have revealed that A/Mdk/6L/07 can spread to several organs, including the heart, spleen, and kidney, in both bird species (Table 2). A/Mdk/6L/07 was also recovered from the brains and spleens of infected mice, whereas LPAI A/Ab/W44/05, a H7N3 virus, was recovered only from the lungs of infected mice, as previously described. We demonstrated that N6 is a required component of trypsin-independent growth and systemic spread and confirmed that this NA-dependent replication mechanism is common to Eurasian N6 lineages rather than being specific to the N6 gene of A/Mdk/6L/07.

The HA proteins of most influenza viruses are cleaved by activated proteases within a membrane-proximal surface loop at an R or K residue adjacent to the G residue that constitutes the N terminus of the newly generated and highly conserved fusion peptide of the HA_2_ subunit. In low-pathogenicity viruses, the cleavage site commonly consists of a monobasic R/K residue, which may be cleaved by trypsin-like serine proteases for activation. An SAP analysis of recently discovered Eurasian H7 genes, including the avian H7N9 viruses that recently infected humans in China, revealed that the H7 influenza viruses contain highly conserved proline, lysine, and glycine residues at the P4, P3, and P2 positions of the cleavage site, respectively, with a conserved arginine at the P1 position that can be recognized and potentially cleaved by thrombin (GRG) (9, 16). Thrombin cleavage motifs that connect peptides in the HA genes of avian influenza species are not commonly found. A comparison with published sequences, including sequences in the GenBank database, showed that the thrombin cleavage motif in HA is highly conserved in, and exclusive to, Eurasian lineages of H7 viruses, including A/Mdk/6L/07 and A/Ab/W44/05 isolates and recent human infectious H7N9 viruses (e.g., A/Anhui/1/2013) (see Appendix and Fig. S3A to C in the supplemental material). Therefore, we hypothesized that the specific HA cleavage motif (GRG) present in A/Mdk/6L/07 is associated with the trypsin-independent replication of the virus and with the N6 neuraminidase. Our recombinant PR/8/34 viruses bearing the modified GRG HA cleavage motif without (PR8_S338G_ [H1N1]) or with PR8_S338G-N6_ (H1N6), the N6 gene segment of A/Mdk/6L/07, were used to confirm that the H1N6 virus had acquired the ability to replicate even in the absence of trypsin and that the PR8_S338G_ (H1N1) strain, which contained a modified HA cleavage motif alone, could not grow as well as the wild-type virus in the absence of trypsin (Fig. 3C and D).

We demonstrated that the dual modification of HA cleavage sites (GRG) along with the N6 NA subtype in various avian influenza A subtypes (H4N6, H5N6, H6N6, H10N6, H11N6, and H12N6) could alter their growth properties to make them trypsin independent in MDCK cells. These results strongly support the idea that the novel replication mechanism used by the A/Mdk/6L/07 virus is not specifically limited to the H7 subtype but could be a general mechanism inherent in many of the avian influenza A viruses. The monobasic cleavage motifs in the HA proteins of the A/WSN/33 and 1918 pandemic H1N1 viruses were originally shown to be cleaved by plasmin after the activation of serum plasminogen in MDBK cells (23), and the virulence properties of these viruses were subsequently linked to the neuraminidase (NA) gene (23, 24). Although the monobasic motifs of H7 subtypes (GRG) have only one amino acid difference from that of A/WSN/33 (SRG), aprotinin and leupeptin (broad-spectrum serine protease inhibitors that efficiently block the replication of A/WSN/33 in the absence of trypsin [14]) failed to affect the replication of the A/Mdk/6L/07 virus in media lacking trypsin (Fig. 5B). However, treatment with argatroban (a direct thrombin inhibitor) significantly attenuated the replication of A/Mdk/6L/07 *in vitro* and *in vivo* (Fig. 5 and 7). These results clearly demonstrate that both the GRG motif in Eurasian H7 viruses and the N6 neuraminidase play crucial roles in the trypsin-independent growth of LPAI H7N6; however, this trypsin-independent growth differs from that of the WSN/33 virus, which was affected by broad-spectrum serine protease inhibitors.

Further *in vitro* studies revealed that direct incubation of prothrombin with purified H7N6, but not H7N3, virus significantly enhanced prothrombin cleavage (Fig. S4). Therefore, we hypothesized that the prothrombin activation by the N6 neuraminidase during replication could facilitate thrombin-dependent cleavage of the GRG motif leading to trypsin-independent replication and multiorgan spread *in vivo*. However, further mechanistic studies are needed to elucidate the details of protease activation by the N6 neuraminidase and, additionally, to further explore the inherent enzymatic activity of this group of proteins.

Since 2013, human infections by novel avian H7N9 virus strains in China are of increasing concern. Although the viruses still replicate mainly in respiratory organs, both H7N9 and H7N6 viruses can be maintained in poultry or migratory bird species. Because the HA proteins of human infectious H7N9 viruses cluster with Eurasian avian lineages and share the cleavage motif of A/Mdk/6L/07 (H7N6), we cannot exclude the emergence of novel H7 reassortants in nature, especially novel H7N6 viruses, which have the HA and internal genes of human infectious H7N9 viruses and the N6 segment of a Eurasian lineage virus. Moreover, domestic ducks and chickens might serve as an interface between the natural gene pool of avian influenza viruses in wild birds and land-based poultry. Therefore, continuous monitoring and systematic surveillance should be instituted to contain these viruses and to reduce their opportunities for further genetic evolution.

In summary, we found that the amino acids at the P4, P3, and P2 positions of the HA cleavage site, along with the N6-specific NA subtype, were related to the efficiency of HA cleavage, replication, and systemic distribution of H7N6 influenza virus in several animal species. We propose that the identity of the amino acids at the GRG cleavage position and the presence of N6 may be indicators of viruses that could pose a public health risk. Therefore, these findings might aid in the prediction and prevention of pandemics emerging at the human-avian interface.

## MATERIALS AND METHODS

### Ethical treatment.

All animal experiments were performed in accordance with the Guidelines for Animal Use and Care of the Korea Center for Disease Control. The experimental procedures used in this study were approved by the Laboratory Animal Research Center (approval numbers CBNU-IRB-2012-GM01 and CBNUA-074-0904-01), which is a member of the Institutional Animal Care and Use Committee of Chungbuk National University. The experiments were performed in biosafety level 3 (BSL3) facilities in partnership with Bioleaders Corp. (permit no. BLS-ABSL-10-003) and the Zoonotic Research Center of Chungbuk National University (BSL3 permit no. KCDC-14-3-07).

### Viruses.

The human H1N1 viruses A/Puerto Rico/8/1934 (A/PR/8/34) and A/WSN/1933 (A/WSN/33) were obtained from St. Jude Children’s Research Hospital. The H7 viruses (A/Mallard duck/Korea/6L/2007 and A/Aquatic bird/Korea/W44/2005) were isolated from the lung tissue of a mallard duck in 2007 and from a fecal sample from a wild aquatic bird in 2005, respectively. The avian viruses used in this study were isolated from migratory birds and domestic animals during routine surveillance (Tables S3 and S4).

### Plasmids and recombinant virus generation.

The 8 gene segments of A/Mdk/6L/07 (H7N6) were amplified and cloned into the pHW2000 plasmid vector to generate recombinant viruses using a plasmid-based reverse-genetics system (25). We also amplified the N1 to N9 genes and several N6 genes from wild bird isolates (Fig. 2 and Table S3 and S4) by reverse transcription-PCR (RT-PCR) and cloned these genes into the pHW2000 vector. The mutation of influenza A viruses tested in this study was approved by the Institutional Animal Care and Use Committee of Chungbuk National University (approval no. CBNU-IRB-2012-GM01) and the Korea National Institute of Health (KNIH) (approval no. 17-RDM-005). Further, all animal studies were conducted in a BSL3 environment officially licensed by the Korea Center for Disease Control (Bioleaders Corp. [permit no. BLS-ABSL-10-003] and the Research Center of Chungbuk National University [BSL3 permit no. KCDC-14-3-07]). Any work that could be interpreted as gain of function was halted during the U.S. Government’s gain-of-function funding pause, and the laboratory strain A/Puerto Rico/8/34 was used as the recipient strain for thrombin cleavage site insertions.

### Genomic sequencing and phylogenetic analysis.

Sequences were prepared and analyzed as previously described (22). Briefly, the A/Mdk/6L/07 and A/Ab/W44/05 strains were prepared for sequencing by Cosmo Genentech (Seoul, Republic of Korea) using an ABI 373 XL DNA sequencer (Applied Biosystems, Foster City, CA). Sequences were analyzed and compiled using the DNAStar 5.0 software (Madison, WI).

### Replication and tissue distribution in animals.

To assess the pathogenicity of A/Mdk/6L/07 in animals, 5-week-old specific-pathogen-free (SPF) White Leghorn chickens (Cavac Laboratories Co., Ltd., South Korea), 3-week-old domestic ducks (Anas platyrhynchos *domesticus*), and 5-week-old female BALB/c mice (Samtaco, Seoul, South Korea) were intranasally inoculated with 10^6^ EID_50_/ml of virus. A variety of tissues, including lung, brain, spleen, kidney, liver, intestine, and heart, were collected for virologic analysis. The viral titers in the sampled tissues were measured by using EID_50_ assays.

### Receptor-binding assays.

Receptor affinity was determined by using a solid-phase direct virus-binding assay, as previously described (17). In brief, influenza viruses were bound to fetuin-coated plates at 4°C overnight. Biotinylated glycans (α2,3′-sialylated glycans. α2,6′-sialylated glycans, or α2,6′-sialyl lactosamine; Glycotech Corporation, Gaithersburg, MD) were added to influenza virus-coated plates at various dilutions and incubated for an additional 4 h. Glycan binding was detected by adding horseradish peroxidase (HRP)-conjugated streptavidin (Invitrogen, Carlsbad, CA), followed by 3,3',5,5'-tetramethylbenzidine (TMB) substrate (Sigma, St. Louis, MO), and the absorbance at 450 nm was measured by using a Victor3 1420 multilabel counter plate reader (PerkinElmer, MA, USA).

### Protease inhibitors and viral replication *in vitro*.

MDCK cells in 6-well plates were infected with A/Mdk/6L/07 (H7N6), A/Ab/W44/05 (H7N3), A/PR/8/34, A/WSN/33, or recombinant viruses at a multiplicity of infection (MOI) of 0.0001. The cells were incubated with virus at 37°C for 1 h, after which the viral inoculates were replaced with appropriate serum-free medium for MDCK cells. Viral growth rates in cells were measured 3 times in duplicate at 37°C in the presence or absence of tosylsulfonyl phenylalanyl chloromethyl ketone (TPCK)-treated trypsin. Cell culture supernatants were harvested at 12, 24, 48, and 72 h postinfection (hpi) for viral titration on MDCK cells, as specified above. A thrombin inhibitor, argatroban, was used at a concentration of 25 μg/ml (approximately 50 μM) to measure cytopathic effects (CPE) and viral growth kinetics (26); the treatment was repeated every 6 h due to the inhibitor’s short half-life. The serine protease inhibitors aprotinin (Sigma-Aldrich) and leupeptin (Sigma-Aldrich) were used at a concentration of 50 μg/ml (approximate 100 μM) in similar assays.

### Argatroban treatment *in vivo*.

To assess the effect of argatroban in animals, female 5-week-old BALB/c mice (Samtaco, Seoul, South Korea) (*n* = 10/group) were intranasally inoculated with 5 LD_50_/mouse of influenza A/PR/8/34 (H1N1), mouse adapted A/Ab/Korea/Ma44/05 (MaH7N3), or A/Mdk/Korea/Ma6L/07 (MaH7N6) virus. Argatroban (Sigma-Aldrich, St. Louis, MO, USA) was dissolved in dimethyl sulfoxide (DMSO) and injected intraperitoneally into mice at a dose of 4.5 or 9 mg/kg daily. The mice then underwent argatroban intraperitoneal injection twice a day for 3 days.

### HA gene cleavage and Western blot analysis.

MDCK cells were infected with wild-type (A/Mdk/6L/07) or mutant viruses at an MOI of 0.0001 in the presence of 0.5 μg/ml TPCK-treated trypsin, aprotinin, leupeptin, or argatroban in minimal essential medium (MEM) without fetal bovine serum (FBS). A polyclonal mouse anti-HA antibody (experimental mouse serum) from A/Mdk/6L/07 H7N6 was used to detect HA. Goat anti-mouse IgG conjugated to horseradish peroxidase was used as a secondary antibody, and the signal was detected by using chemiluminescence.

### Data availability.

The GenBank accession numbers assigned to the sequences generated in this study are HQ913047, HQ913054, HQ913060, KX297750, KX297798, KX297782, KX297766, and KX297814.
