# Supplementary material for: A Novel Neuraminidase-Dependent Hemagglutinin Cleavage Mechanism Enables the Systemic Spread of an H7N6 Avian Influenza Virus
Source: mBio. 2019 Nov 5;10(6):e02369-19. doi: 10.1128/mBio.02369-19 (PMC6831776; doi:10.1128/mBio.02369-19)
Supplement: TABLE S1 [file mBio.02369-19-st001.docx]

**Supplementary Table 1.** Sequence homology of each gene of Mdk/Korea/6L/07 (H7N6) influenza virus with reference viruses available in GenBank.

| **Gene** | **Virus with the highest homology [accession no.]^a^** | **Nucleotide sequence identity** | **Host** |
| --- | --- | --- | --- |
| **PB2** | A/avian/Japan/8KI0102/2008(H3N8) [CY079266] | 99.3% | Avian |
| **PB1** | A/aquatic bird/Korea/w44/2007(H7N3) [HQ913055] | 99.9% | Avian |
| **PA** | A/aquatic bird/Korea/w44/2007(H7N3) [HQ913057] | 99.9% | Avian |
| **HA** | A/wild duck/Jiangxi/10179/05(H7N3) [KF259000] | 99.3% | Avian |
| **NP** | A/Mallard/Jiangxi/12147/05(H6N2) [HM145193] | 99.9% | Avian |
| **NA** | A/duck/Eastern china/01/07(H4N6)[EU429790] | 98.7% | Avian |
| **M** | A/Mallard/Yan chen/05(H4N6) [EU880343] | 99.6% | Avian |
| **NS** | A/duck/Guizhou/1560/07(H6N8) [CY109654] | 99.6% | Avian |

^a^ The numbers in brackets are GenBank accession numbers for the reference virus.
