# Supplementary figures and images for: A Novel Neuraminidase-Dependent Hemagglutinin Cleavage Mechanism Enables the Systemic Spread of an H7N6 Avian Influenza Virus
Source: mBio. 2019 Nov 5;10(6):e02369-19. doi: 10.1128/mBio.02369-19 (PMC6831776; doi:10.1128/mBio.02369-19)

Supplementary Figure. 1

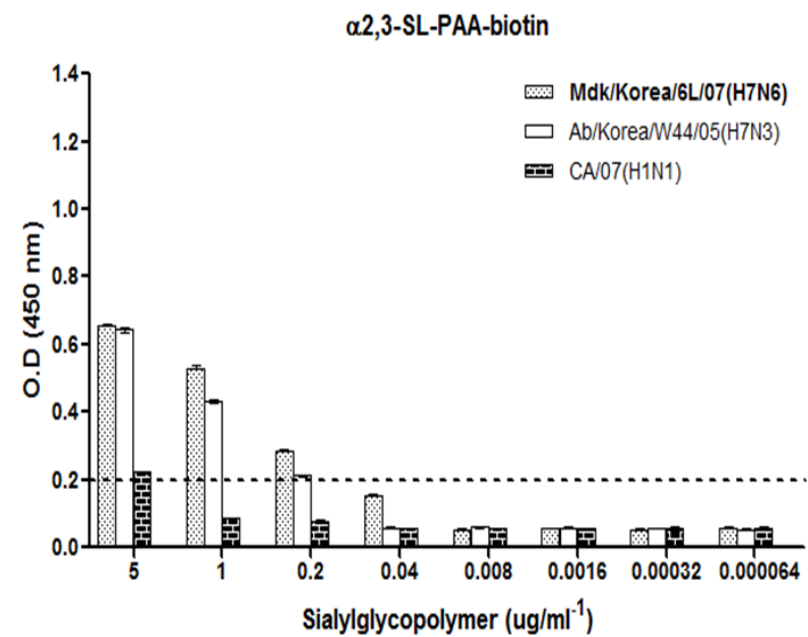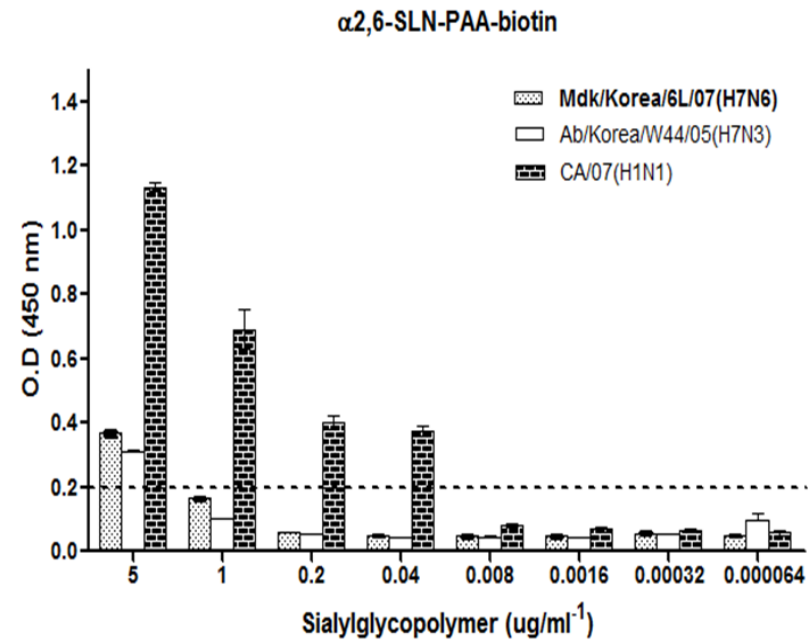

Supplement: FIG S1 [file mBio.02369-19-sf001.pdf]

## Supplementary Figure. 6

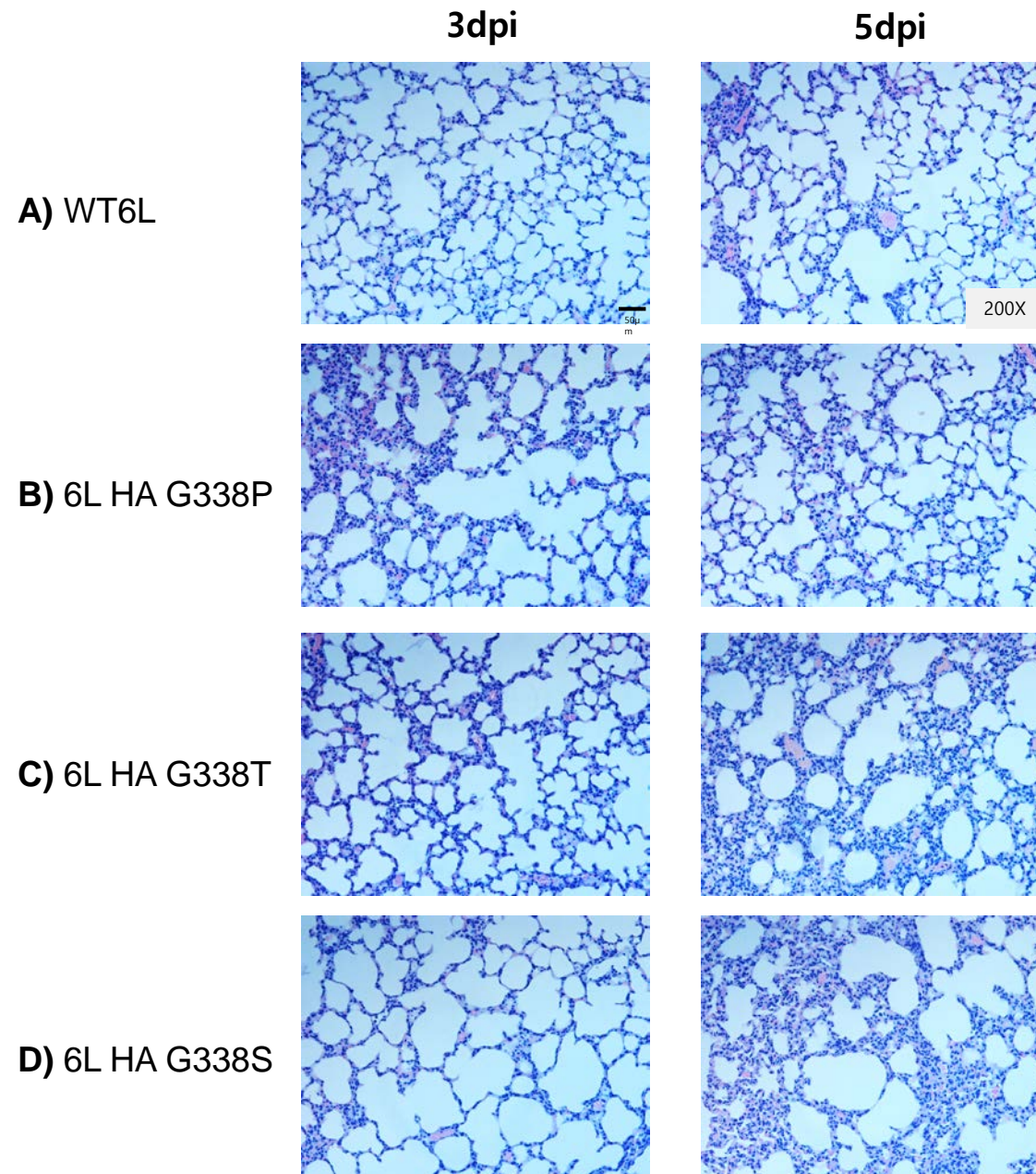

## Supplementary Figure. 6-continue

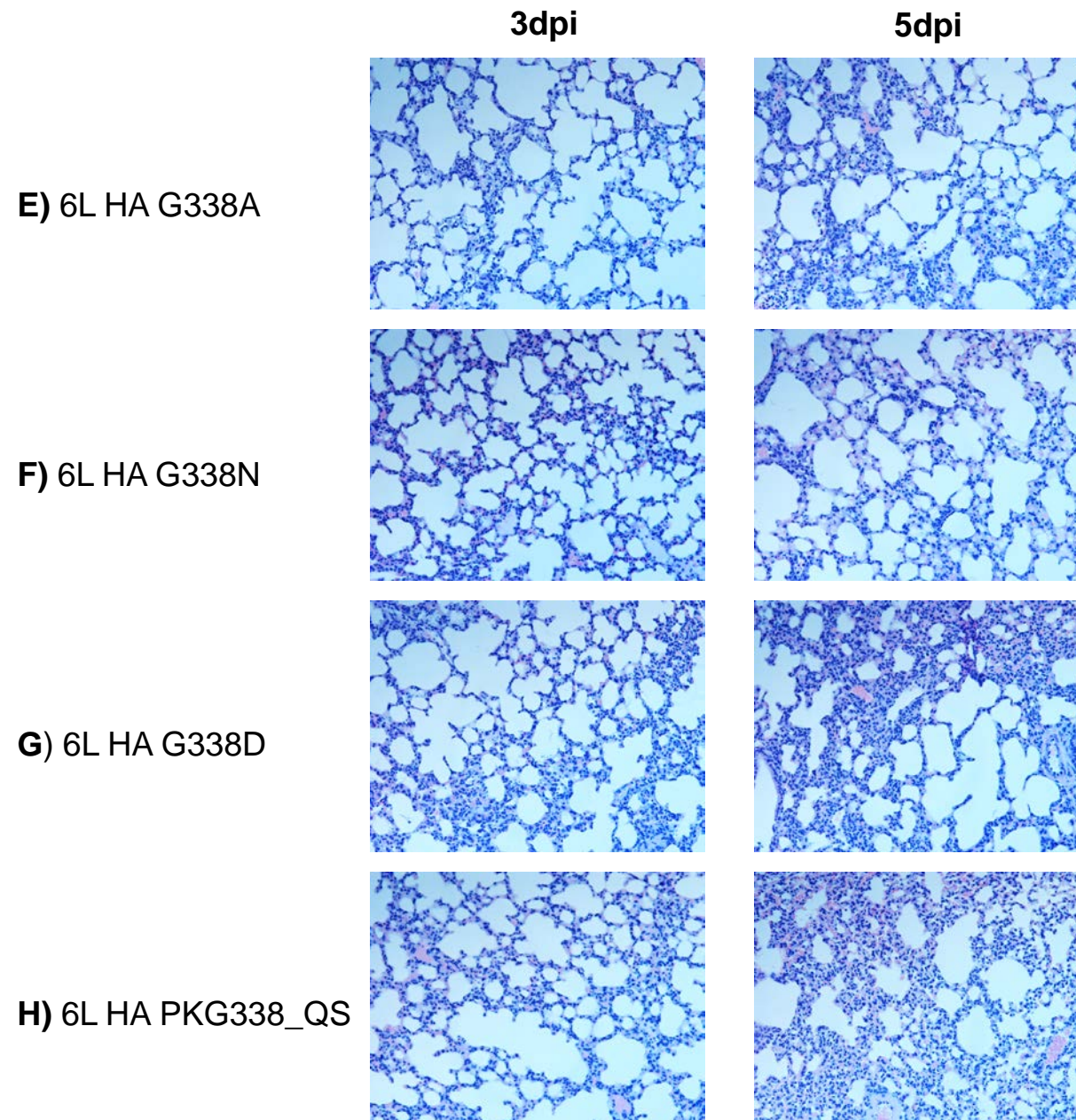

## Supplementary Figure. 6- continue

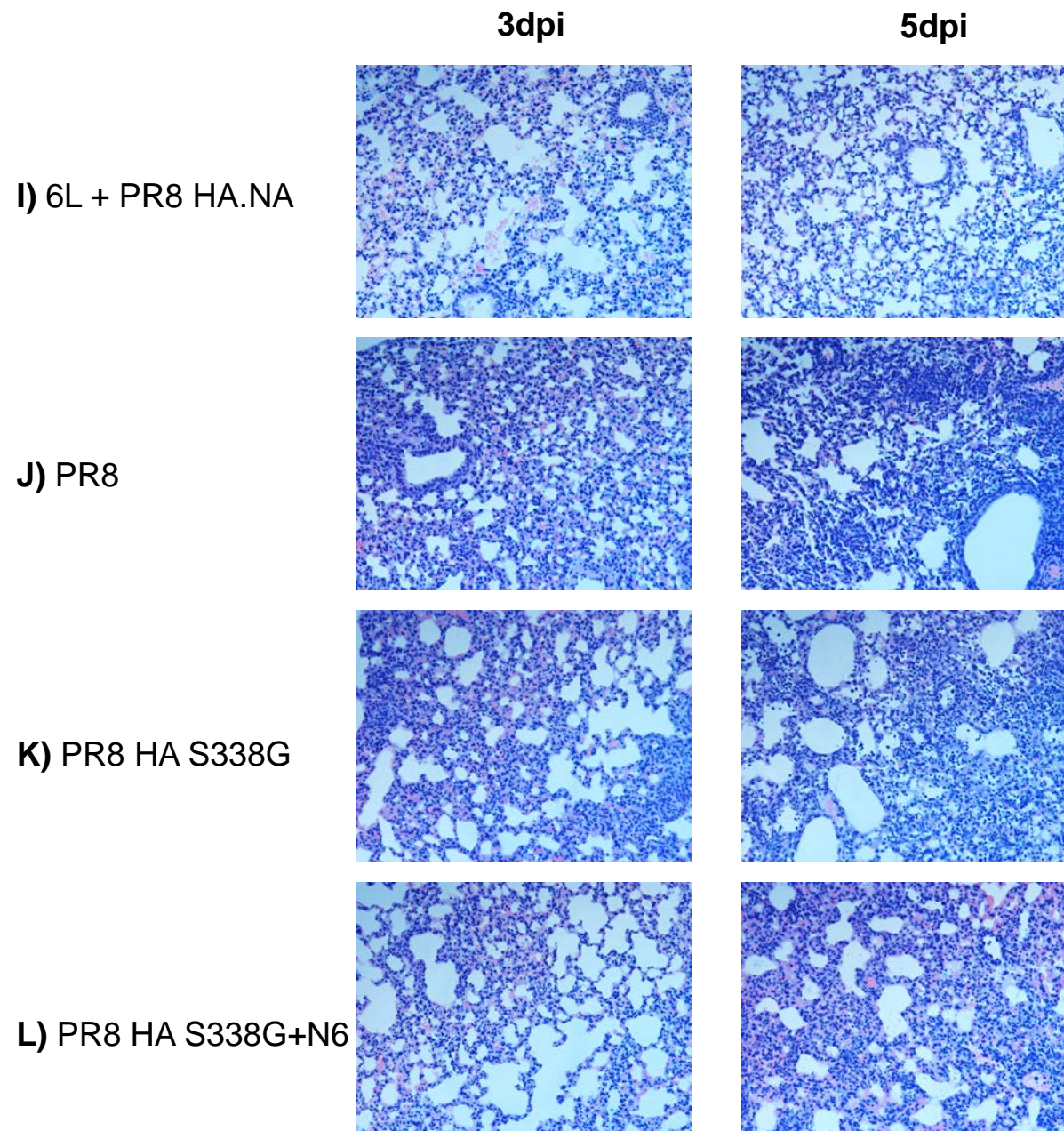

Supplement: FIG S6 [file mBio.02369-19-sf006.pdf]

Figure S2.

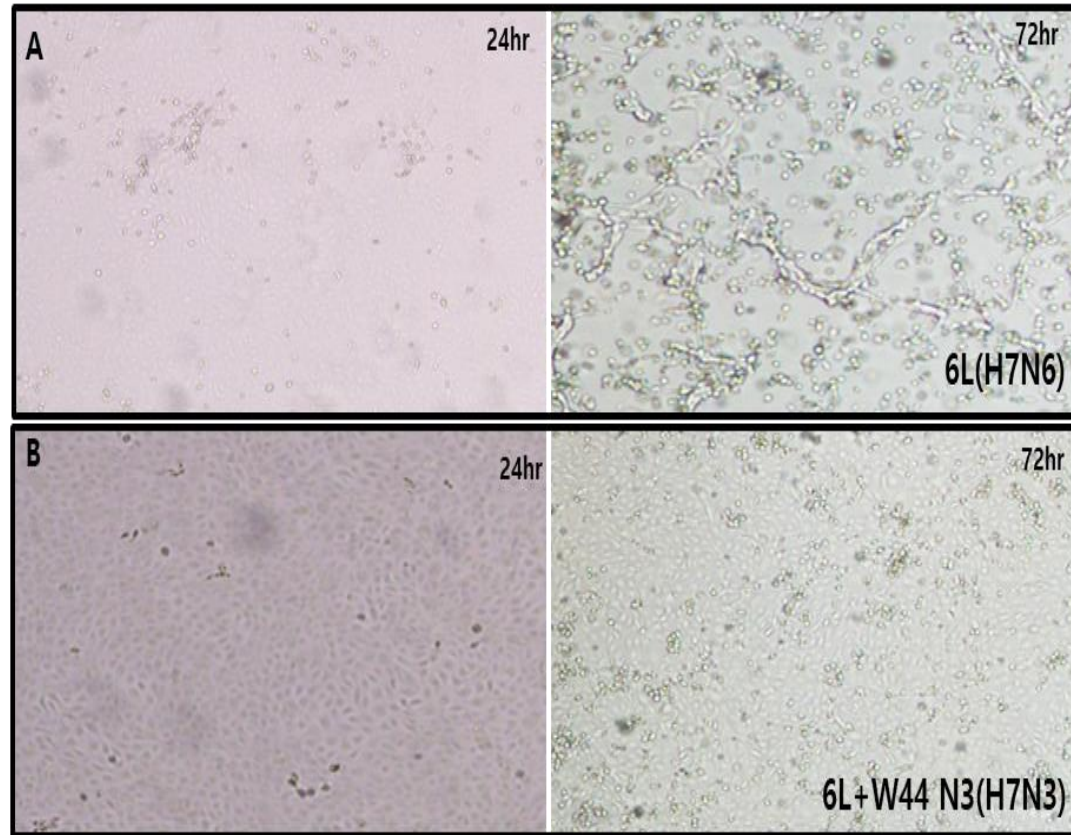

Supplement: FIG S2 [file mBio.02369-19-sf002.pdf]

### Supplementary Figure. 5

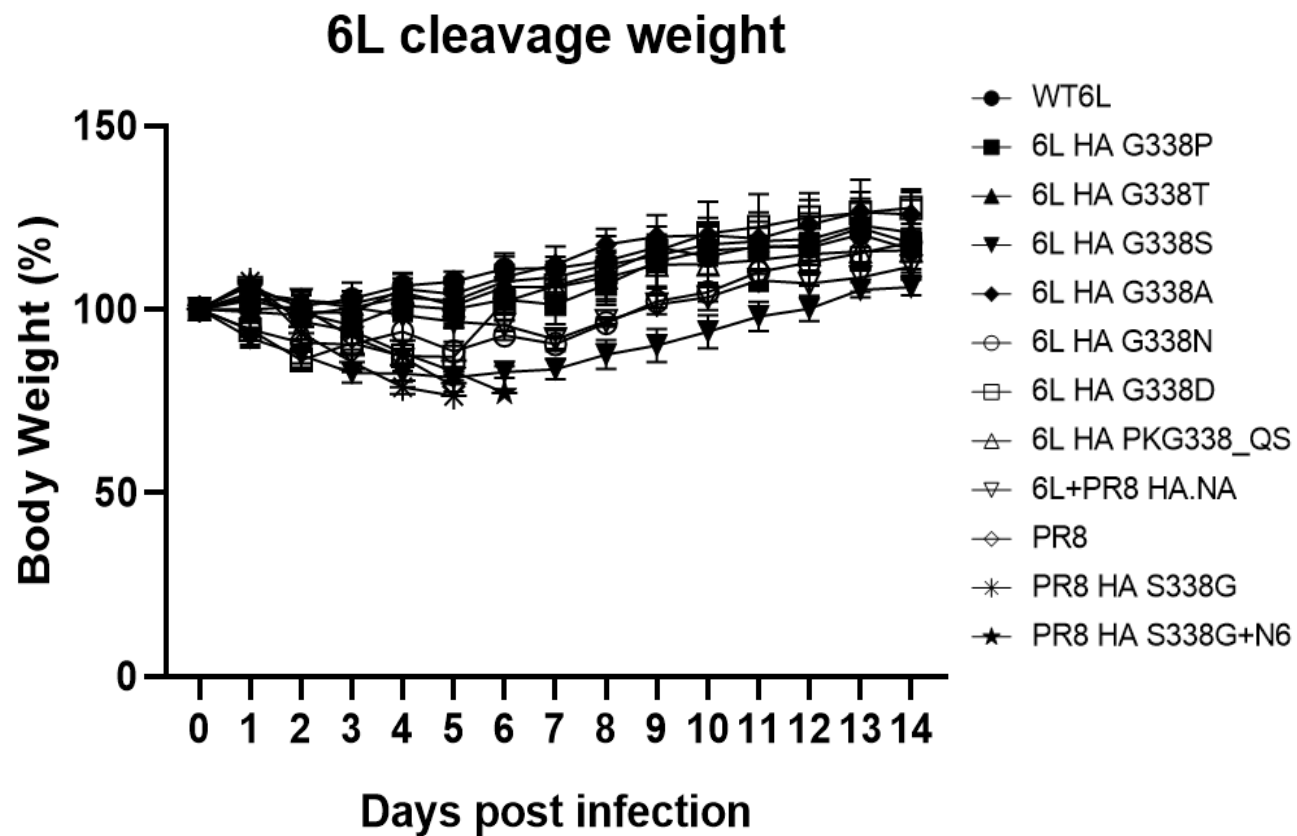

Supplement: FIG S5 [file mBio.02369-19-sf005.pdf]

Supplementary Figure. 4

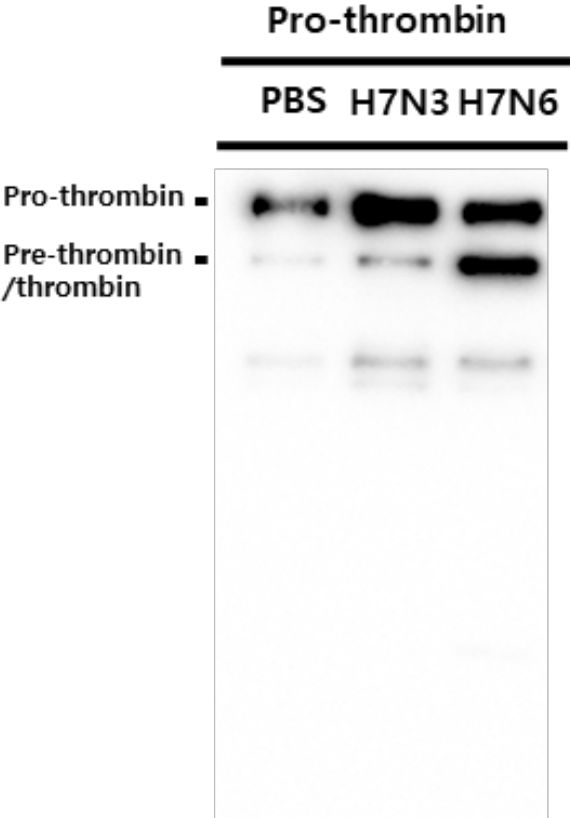

Supplement: FIG S4 [file mBio.02369-19-sf004.pdf]
