# Supplementary material for: A Novel Neuraminidase-Dependent Hemagglutinin Cleavage Mechanism Enables the Systemic Spread of an H7N6 Avian Influenza Virus
Source: mBio. 2019 Nov 5;10(6):e02369-19. doi: 10.1128/mBio.02369-19 (PMC6831776; doi:10.1128/mBio.02369-19)
Supplement: TABLE S2 [file mBio.02369-19-st002.docx]

**Supplementary Table 2.** Viral titer and MLD_50_ of a modified HA cleavage site in Md/Korea/6L/07(H7N6).

| **Virus** | **Titer (Log_10_EID_50_/ml)** | **MLD_50_ (Log_10_EID_50_/ml)** |
| --- | --- | --- |
| **WT6L** | 8.7^a^ | > 8.7 |
| **6L HA G338P** | 8.2 | > 8.2 |
| **6L HA G338T** | 9.2 | > 9.2 |
| **6L HA G338S** | 9.7 | > 9.7 |
| **6L HA G338A** | 9.7 | > 9.7 |
| **6L HA G338N** | 8.5 | > 8.5 |
| **6L HA G338D** | 9.2 | > 9.2 |
| **6L HA PKG338_QS** | 9.7 | > 9.7 |
| **6L + PR8 HA.NA** | 6.7 | > 6.7 |
| **PR8** | 7.4 | 4.9 |
| **PR8 HA S338G** | 9.2 | 4.7 |
| **PR8 HA S338G+N6** | 8.2 | 5.2 |

^a^EID_50_ values were measured in 12-day-old embryonated chicken eggs.
