# Supplementary material for: A Novel Neuraminidase-Dependent Hemagglutinin Cleavage Mechanism Enables the Systemic Spread of an H7N6 Avian Influenza Virus
Source: mBio. 2019 Nov 5;10(6):e02369-19. doi: 10.1128/mBio.02369-19 (PMC6831776; doi:10.1128/mBio.02369-19)
Supplement: TABLE S3 [file mBio.02369-19-st003.docx]

**Supplementary Table 3.** Viral replication of HA- and NA-substituted viruses in the genetic backbone of 6L virus

| **Recombinant Virus Name** | **Generated Subtype** | **Substituted Gene** | | **Parental Virus Subtype** | **Titration**  **log_10_TCID_50_/ml (±SD)** | |
| --- | --- | --- | --- | --- | --- | --- |
|  |  |  |  |  | **Trypsin** | **w/o Trypsin** |
| RG6L | H7N6 | NC | Mdk/Korea/6L/07(H7N6) | | 6.75(0.3)^a^ | 6.25(0.3) |
| **Various HA Subtype** | | | | | | |
| RG44 | H7N6 | HA(H7) | Ab/Korea/W44/05(H7N3) | | 6.5(0.1) | 6.25(0.3) |
| RGPR8 | H1N6 | HA(H1) | A/Puerto Rico/8/34(H1N1) | | 8.25(0.3) | 7.25(0.3) |
| RGL91 | H3N6 | HA(H3) | Dk/Korea/L91/07(H3N2) | | 7.25(0.3) | 2.5(0.1) |
| RG81 | H5N6 | HA(H5) | Ab/Korea/W81/05(H5N2) | | 7.25(0.3) | 2.25(0.3) |
| RG164 | H9N6 | HA(H9) | Ck/Korea/L164/09(H9N2) | | 5.5(0.1) | 2.25(0.3) |
|  |  |  |  | |  |  |
| **Various NA subtype** | | | | | | |
| NRG24 | H7N1 | NA(N1) | Ab/Korea/W24/05(H6N1) | | 1.5(0.1) | 1.25(0.3) |
| NRG82 | H7N2 | NA(N2) | Ab/Korea/W82/05(H7N2) | | 3.25(0.3) | 1.25(0.3) |
| NRG16 | H7N3 | NA(N3) | Ab/Korea/W16/05(H5N3) | | 6.25(0.3) | 1.75(0.3) |
| NRG123 | H7N4 | NA(N4) | Ab/Korea/W123/05(H7N4) | | 7.5(0.1) | 2.25(0.3) |
| NRG69 | H7N5 | NA(N5) | Ab/Korea/W69/05(H6N5) | | 6.25(0.3) | 1.75(0.3) |
| NRG184 | H7N7 | NA(N7) | Ab/Korea/W184/05(H4N7) | | 5.25(0.3) | 1.5(0.1) |
| NRG06 | H7N8 | NA(N8) | Ab/Korea/W06/05(H6N8) | | 5.25(0.3) | 1.5(0.1) |
| NRG118 | H7N9 | NA(N9) | Ab/Korea/W118/06(H2N9) | | 6.25(0.3) | 1.75(0.3) |
|  |  |  |  | |  |  |
| **Various N6 gene** | | | | | | |
| NRG20 | H7N6 | NA(N6) | Ab/Korea/W20/05(H4N6) | | 6.5(0.1) | 6.25(0.3) |
| NRG111 | H7N6 | NA(N6) | Ab/Korea/W111/06(H4N6) | | 6.25(0.3) | 6.25(0.3) |
| NRG119 | H7N6 | NA(N6) | Ab/Korea/W119/06(H4N6) | | 6.25(0.3) | 5.75(0.3) |
| NRG223 | H7N6 | NA(N6) | Ab/Korea/W223/07(H6N6) | | 6.25(0.3) | 6.25(0.3) |
| NRG337 | H7N6 | NA(N6) | Ab/Korea/W337/08(H4N6) | | 7.25(0.3) | 6.75(0.3) |
| NRGL38 | H7N6 | NA(N6) | Dk/Korea/L38/05(H3N6) | | 6.25(0.3) | 6.25(0.3) |

NC; No change in gene

^a^Standard deviation titers

^b^Sample titration performed at 72 h.

Mdk, Mallard duck; Ab, Aquatic bird; Dk, Duck; Ck, chicken; Gf, guinea fowl.
