# Supplementary material for: A Novel Neuraminidase-Dependent Hemagglutinin Cleavage Mechanism Enables the Systemic Spread of an H7N6 Avian Influenza Virus
Source: mBio. 2019 Nov 5;10(6):e02369-19. doi: 10.1128/mBio.02369-19 (PMC6831776; doi:10.1128/mBio.02369-19)
Supplement: FIG S3 [file mBio.02369-19-sf003.pdf]

# Supplementary Figure. 3

## A

Cleavage site (between HA1 and HA2)

| | | | | | HA1 P4 | P3 | P2 | P1 | HA2 P1' | | | | | | | | | | | | |
|---|---|---|---|---|---|---|---|---|---|---|---|---|---|---|---|---|---|---|---|---|---|
| Eurasian H7 consensus | - | P | E | I | **P** | **K** | **G** | **R** | **G** | L | F | G | A | I | A | G | F | I | E | N | G |
| A/Mdk/Korea/6L/07 | | · | · | · | • | • | • | • | • | · | · | · | · | · | · | · | · | · | · | · | · |
| A/Ab/Korea/W44/05 | | · | · | · | • | • | • | • | • | · | · | · | · | · | · | · | · | · | · | · | · |
| A/Mdk/Korea/GH171/07 | | · | · | · | • | • | • | • | • | · | · | · | · | · | · | · | · | · | · | · | · |
| A/Tk/Italy/9739/02 | | · | · | · | • | • | • | • | • | · | · | · | · | · | · | · | · | · | · | · | · |
| A/Mdk/Nl/12/00 | | · | · | · | • | • | • | • | • | · | · | · | · | · | · | · | · | · | · | · | · |
| A/Anhui/1/13 | | · | · | · | • | • | • | • | • | · | · | · | · | · | · | · | · | · | · | · | · |
| A/Shanghai/2/13 | | · | · | · | • | • | • | • | • | · | · | · | · | · | · | · | · | · | · | · | · |
| A/Tk/Minnesota/18421/09 | | · | · | · | • | • | T | • | • | · | · | · | · | · | · | · | · | · | · | · | · |
| A/Ck/DE/HOBO/04 | | · | · | K | • | • | P | • | • | · | · | · | · | · | · | · | · | · | · | · | · |
| H1 consensus | - | P | S | **I** | **-** | **Q** | **S** | **R** | **G** | L | F | G | A | I | A | G | F | I | E | N | G |
| A/PR/8/34 | | · | · | • | - | • | • | • | • | · | · | · | · | · | · | · | · | · | · | · | · |
| A/WSN/33 | | · | · | • | - | • | • | • | • | · | · | · | · | · | · | · | · | · | · | · | · |
| A/California/04/09 | | · | · | • | - | • | **Y** | • | • | · | · | · | · | · | · | · | · | · | · | · | · |

## B

| P2 position | Rate (%) | Lineage | Isolated Nation |
|---|---|---|---|
| G | 39.1 | Eurasian | South Korea, China, Japan<br>Hong Kong, Thailand, Egypt<br>Spain, Germany, England |
| T | 34 | American | USA, Canada, Chile |
| P | 18.9 | | Mexico, New York |
| R, K | 8 | | New York, Netherlands |

## C

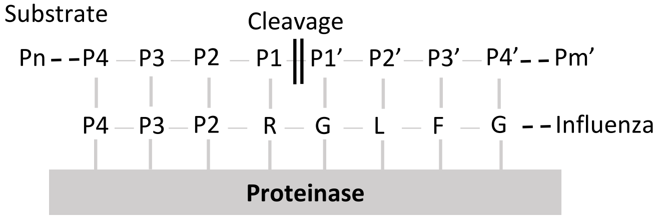


| Enzyme name | P4 | P3 | P2 | P1 | P1' | P2' |
|---|---|---|---|---|---|---|
| **Trypsin** | - | - | - | K or R | not P | - |
| | - | - | W | K | P | - |
| | - | - | M | R | P | - |
| **Thrombin** | **-** | **-** | **G** | **R** | **G** | **-** |
| | A,F,G,I, L,T,V,M | A,F,G,I,L, T,V,W,A | P | R | not D or E | not DE |
