# Supplementary material for: A Novel Neuraminidase-Dependent Hemagglutinin Cleavage Mechanism Enables the Systemic Spread of an H7N6 Avian Influenza Virus
Source: mBio. 2019 Nov 5;10(6):e02369-19. doi: 10.1128/mBio.02369-19 (PMC6831776; doi:10.1128/mBio.02369-19)
Supplement: TABLE S4 [file mBio.02369-19-st004.docx]

**Supplementary Table 4.** Viral replication of HA- and NA-substituted viruses in the genetic backbone of 6L and PR8 virus

| **Recombinant Virus Name** | **Rescued** | | | **Generated Subtype** | **Substituted Gene Segment** | | | **Parental Virus Subtype** | **Titration**  **log_10_TCID_50_/ml (±SD)** | |
| --- | --- | --- | --- | --- | --- | --- | --- | --- | --- | --- |
|  |  |  |  |  |  |  |  |  | **Trypsin** | **w/o Trypsin** |
| RG6L | | Y | | H7N6 | | NC | Mdk/Korea/6L/07(H7N6) | | 6.75(0.3)^a^ | 6.25(0.3) |
| **PR8 backbone** | | | | | | | | | | |
| H1_GRG_+N6 | | N | H1N6 | | | HA(H1), NA(N6) | California/07/09(H1N1) | | ND | ND |
| H2_GRG_+N6 | | N | H2N6 | | | HA(H2), NA(N6) | Ab/Korea/W357/08(H2N3) | | ND | ND |
| H3_GRG_+N6 | | N | H3N6 | | | HA(H3), NA(N6) | Perth/16/09 | | ND | ND |
| H4_GRG_+N6 | | Y | H4N6 | | | HA(H4), NA(N6) | Ab/Korea/W360/08(H4N6) | | 6.25(0.3) | 4.75(0.5) |
| H5_GRG_+N6 | | Y | H5N6 | | | HA(H5), NA(N6) | Ab/Korea/W81/05(H5N2) | | 6.75(0.3) | 4.25(0.3) |
| H6_GRG_+N6 | | Y | H6N6 | | | HA(H6), NA(N6) | Ab/Korea/W340/08(H6N6) | | 7.25(0.3) | 3.47(0.5) |
| H8_GRG_+N6 | | N | H8N6 | | | HA(H8), NA(N6) | Ab/Korea/W141/06(H8N4) | | ND | ND |
| H9_GRG_+N6 | | N | H9N6 | | | HA(H9), NA(N6) | Ck/Korea/L164/09(H9N2) | | ND | ND |
| H10_GRG_+N6 | | Y | H10N6 | | | HA(H10), NA(N6) | Dk/Korea/7T/07(H10N4) | | 7.25(0.5) | 4.75(0.5) |
| H11_GRG_+N6 | | Y | H11N6 | | | HA(H11), NA(N6) | Ab/Korea/W423/12(H11N9) | | 6.75(0.3) | 4.75(0.3) |
| H12_GRG_+N6 | | Y | H12N6 | | | HA(H12), NA(N6) | Ab/Korea/W424/12(H12N5) | | 6.75(0.3) | 3.75(0.5) |
|  | |  |  | | |  |  | |  |  |
| **WT6L backbone** | | | | | | | | | | |
| H1_GRG_+N6 | | N | H1N6 | | | HA(H1) | California/07/09(H1N1) | | ND | ND |
| H2_GRG_+N6 | | N | H2N6 | | | HA(H2) | Ab/Korea/W357/08(H2N3) | | ND | ND |
| H3_GRG_+N6 | | N | H3N6 | | | HA(H3) | Perth/16/09 | | ND | ND |
| H4_GRG_+N6 | | Y | H4N6 | | | HA(H4) | Ab/Korea/W360/08(H4N6) | | 6.75(0.3) | 6.25 |
| H5_GRG_+N6 | | Y | H5N6 | | | HA(H5) | Ab/Korea/W81/05(H5N2) | | 6.75(0.5) | 6.25(0.3) |
| H6_GRG_+N6 | | Y | H6N6 | | | HA(H6) | Ab/Korea/W340/08(H6N6) | | 7.25(0.3) | 6.5(0.5) |
| H8_GRG_+N6 | | N | H8N6 | | | HA(H8) | Ab/Korea/W141/06(H8N4) | | ND | ND |
| H9_GRG_+N6 | | N | H9N6 | | | HA(H9) | Ck/Korea/L164/09(H9N2) | | ND | ND |
| H10_GRG_+N6 | | Y | H10N6 | | | HA(H10) | Dk/Korea/7T/07(H10N4) | | 7.5(0.3) | 6.75(0.5) |
| H11_GRG_+N6 | | Y | H11N6 | | | HA(H11) | Ab/Korea/W423/12(H11N9) | | 6.75(0.3) | 6.25(0.3) |
| H12_GRG_+N6 | | Y | H12N6 | | | HA(H12) | Ab/Korea/W424/12(H12N5) | | 6.75(0.5) | 6.25(0.5) |

NC, No change in gene; ND, Not determined; Y, Yes; N, No

^a^Standard deviation titers

^b^Sample titration performed at 72 h.

Mdk, Mallard duck; Ab, Aquatic bird; Dk, Duck; Ck, chicken.
